# Identification of a genomic enhancer that enforces proper apoptosis induction in thymic negative selection

**DOI:** 10.1038/s41467-019-10525-1

**Published:** 2019-06-13

**Authors:** Miki Arai Hojo, Kyoko Masuda, Hiroaki Hojo, Yosuke Nagahata, Keiko Yasuda, Daiya Ohara, Yusuke Takeuchi, Keiji Hirota, Yutaka Suzuki, Hiroshi Kawamoto, Shinpei Kawaoka

**Affiliations:** 1 Graduate School of Frontier Science, The University of Tokyo, Kashiwa-shi, Chiba, 277-8562 Japan; 2 The Thomas N. Sato BioMEC-X Laboratories, Advanced Telecommunications Research Institute International (ATR), Soraku-gun, Kyoto, 619-0237 Japan; 3 Institute for Frontier Life and Medical Sciences, Kyoto University, Kyoto-shi, Kyoto, 606-8507 Japan; 4 ERATO Sato Live Bio-forecasting Project, Japan Science and Technology Agency (JST), Soraku-gun, Kyoto, 619-0237 Japan

**Keywords:** Lymphocyte differentiation, Central tolerance, Epigenetics in immune cells, Immune cell death

## Abstract

During thymic negative selection, autoreactive thymocytes carrying T cell receptor (TCR) with overtly strong affinity to self-MHC/self-peptide are removed by Bim-dependent apoptosis, but how *Bim* is specifically regulated to link TCR activation and apoptosis induction is unclear. Here we identify a murine T cell-specific genomic enhancer *E*^*BAB* (*Bub1*-*Acoxl*-*Bim*)^, whose deletion leads to accumulation of thymocytes expressing high affinity TCRs. Consistently, *E*^*BAB*^ knockout mice have defective negative selection and fail to delete autoreactive thymocytes in various settings, with this defect accompanied by reduced *Bim* expression and apoptosis induction. By contrast, *E*^*BAB*^ is dispensable for maintaining peripheral T cell homeostasis via Bim-dependent pathways. Our data thus implicate *E*^*BAB*^ as an important, developmental stage-specific regulator of *Bim* expression and apoptosis induction to enforce thymic negative selection and suppress autoimmunity. Our study unravels a part of genomic enhancer codes that underlie complex and context-dependent gene regulation in TCR signaling.

## Abstract (web summary)

Autoreactive T cells are deleted in the thymus via thymic negative selection and Bim-mediated apoptosis. Here the authors identify a *cis*-acting enhancer, *E*^*BAB*^, that is essential for proper *Bim* expression and apoptosis induction, and show that *E*^*BAB*^ deficiency specifically impairs thymic negative selection without affecting peripheral T cell homeostasis.

## Introduction

T cell population in the thymus is highly heterogeneous, harboring a diverse T cell receptor (TCR) repertoire^1–4^. The massive diversity in TCR sequences is on one hand useful, as it puts the immune system on stand-by for numerous foreign antigens such as pathogens, while on the other hand it is risky because it could generate T cells harboring TCRs that strongly recognize self, potentially causing autoimmunity. To suppress autoimmunity, organisms have evolved a sophisticated mechanism called negative selection, establishing central T cell tolerance. In negative selection, interaction between TCR and self-peptide presented on major histocompatibility complexes (self-pMHCs) is converted into apoptotic output: high-affinity TCR clones are considered as autoreactive and die by apoptosis^1–4^.

A part of high-affinity TCR clones, instead of being deleted, are diverted into regulatory T (T_reg_) cells that are potent to suppress autoreactive T cells in periphery^1,5^. Suppression by T_reg_ cells is one of the peripheral tolerance mechanisms for organisms to deal with autoreactive T cells that have evaded negative selection. Other mechanisms are induction of T cell anergy and peripheral deletion by apoptosis^1^.

Pro-apoptotic Bim promotes the mitochondrial apoptosis cascade, contributing to numerous biological pathways^6–18^. In central T cell tolerance, *Bim* is considered as a downstream target of TCR signal: TCR signal activates *Bim* expression, and *Bim* knockout (KO) mice show defective negative selection^6^. However, little is known about how TCR signal strength is linked to *Bim* expression^2,17^.

*Bim* is genetically required not only for establishing central T cell tolerance^6–8^, but also for depleting activated T cells in periphery^11,12^, B cell homeostasis, embryonic development, and so on^18^. Therefore, *Bim* should be able to distinguish multiple biological pathways in different cell types, depending on signals that cells receive. The molecular mechanism underlying how *Bim* is regulated to work at an appropriate place and time remains elusive.

Enhancers are genomic elements that regulate gene expression in a signal and cell type dependent manner^19,20^. Although epigenome analyses have enabled systematic identification and characterization of enhancers, it is still difficult to directly study their physiological roles in vivo for the following reasons. First, enhancers are located often several hundreds of kilobases to even megabases away from their target genes, making it difficult to confidently predict a target(s) of an enhancer. Second, some genes may have multiple functionally redundant enhancers. Third, making enhancer KOs through genetic ablation has been labor-intensive and time-consuming, especially in mice. Recent progress in CRISPR–Cas9 technology^21^ has reduced the cost and time needed for generating enhancer KO mice, and most importantly, has enabled us to produce large genomic deletions without leaving unwanted footprints of exogenous DNAs. CRISPR–Cas9 technology is indeed beginning to uncover physiological functions of novel enhancers in vivo^22–24^.

Here, we utilize enhancer genetics to understand how *Bim* is specifically regulated to induce apoptosis during thymic negative selection, and find a *cis*-regulatory enhancer specifically contributing to this process. With the aid of epigenome analyses, we identify a *cis*-regulatory enhancer *E*^*BAB* (*Bub1*-*Acoxl*-*Bim*)^ that is specific to thymocytes and splenic T cells. We generate *E*^*BAB*^ KO mice by CRISPR–Cas9 technology and find that a high-affinity TCR repertoire accumulates in the *E*^*BAB*^ KO thymus. *E*^*BAB*^ KO thymocytes are defective in apoptosis due to incomplete activation of *Bim*. By contrast, *Bim*-mediated homeostasis of T_reg_ cells and peripheral T cells is not affected by *E*^*BAB*^ KO, thereby implicating a specific function of *E*^*BAB*^ in thymic negative selection. This study is an example of utilizing enhancer KO approach to dissect regulation of enhancer activity and subsequent gene function in vivo to address biological questions.

## Results

### Identification of a murine T cell-specific enhancer *E*^*BAB*^

**Fig. 1:**
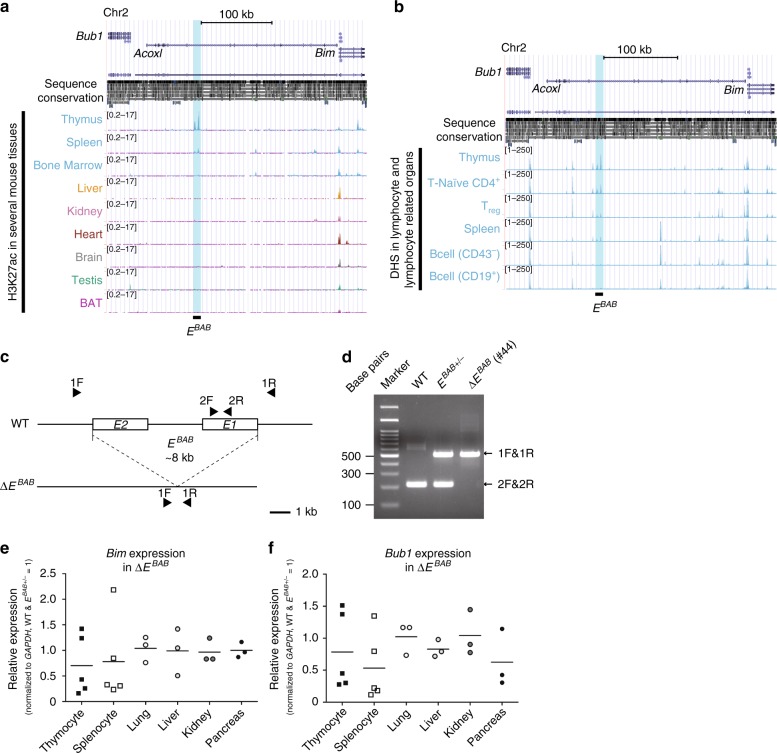
Identification of a T cell-specific *cis*-regulatory element *E*^*BAB*^. **a** ChIP-seq visualization of H3K27ac in several mouse tissues. H3K27ac profiles from the thymus, spleen, bone marrow, liver, kidney, heart, brain, testis, and brown adipose tissues (BAT) are visualized using the UCSC genome browser (mm9). The *E*^*BAB*^ region is highlighted. **b** DNase hypersensitivity sites (DHS) in the same locus shown in (**a**). DHS profiles from the thymus, T-Naïve CD4^+^, regulatory T (T_reg_) cells, spleen, and B cells (CD43^−^ or CD19^+^) are visualized using the UCSC genome browser (mm9). The *E*^*BAB*^ region is highlighted. **c** Schematic representation of Δ*E*^*BAB*^. Arrowheads indicate the primers listed in Supplementary Data 1. **d** Genomic PCR against the *E*^*BAB*^ locus of WT, heterozygotes (*E*^*BAB*+/−^), and Δ*E*^*BAB*^ mice. A representative gel-image of founder #44-derived DNAs is shown. See also Supplementary Fig. 2 for the results from #47- and #50-derived DNAs. **e**, **f** qPCR analysis for *Bim* (**e**) and *Bub1* (**f**) on thymocytes, splenocytes, lung, liver, kidney, and pancreas. Data are pooled from five independent experiments (thymocyte, splenocyte; *n* = 5 WT and *E*^*BAB*+/−^–Δ*E*^*BAB*^ littermate pairs, 7–17 weeks old, mean ± s.d.) or three independent experiments (lung, liver, kidney, pancreas; *n* = 3 WT & *E*^*BAB*+/−^–Δ*E*^*BAB*^ littermate pairs, 10–17 weeks old). Each symbol represents an individual mouse; small horizontal lines indicate the mean. No statistically significant differences between WT and *E*^*BAB*+/−^ and Δ*E*^*BAB*^ were detected (*P* ≥ 0.05; unpaired two-tailed Student’s *t* test)

Our analyses on various publicly available ChIP-seq (chromatin-immunoprecipitation and following sequencing) data on multiple mouse tissues identified a T cell-specific enhancer-like region (H3K27ac high and H3K4me3 low)^19^ in the mouse *Bub1*-*Acoxl*-*Bim* locus (Fig. 1a and Supplementary Fig. 1). This region was located at approximately 200-kb upstream of *Bim*, within the ninth intron of *Acoxl* gene (unexpressed in T cells), and at approximately 90-kb upstream of *Bub1* (a mitotic checkpoint factor), and thus was named *E*^*BAB*^. *E*^*BAB*^ was approximately 8-kb in length and contained two prominent H3K27ac peaks *E1* and *E2* (Fig. 1a). Both *E1* and *E2* were highly specific to the thymus (Fig. 1a) and well conserved between human and mice (Fig. 1a and Supplementary Fig. 1b). H3K27ac peaks corresponding to *E*^*BAB*^ were identified also in the spleen to a lesser extent (Fig. 1a). The signals in the spleen were likely derived from splenic peripheral T cells because naïve peripheral T cells, but not CD19^+^ B cells, retained DNase hypersensitivity sites in the locus (Fig. 1b), the observation further supported by other publicly available ChIP-seq data sets (Supplementary Fig. 1c).

To investigate a physiological role of *E*^*BAB*^ in vivo, we generated *E*^*BAB*^ KO mice by using the CRISPR–Cas9 system (Fig. 1c and Supplementary Data 1). Three founder lines (line#44, #47 and #50) were successfully obtained, each harboring a distinct pattern of deletion (Fig. 1d and Supplementary Fig. 2). Because offspring from these different founders showed no phenotypic differences (Supplementary Fig. 3), we refer to these three KO alleles simply as Δ*E*^*BAB*^ in this paper.

To ask if *E*^*BAB*^ KO affects expression of *Bim* and *Bub1*, we performed quantitative polymerase chain reaction (qPCR) experiments on several organs and cell types including thymocytes and splenocytes (Fig. 1e, f). *Bim* and *Bub1* expression were slightly decreased in thymocytes and splenocytes in Δ*E*^*BAB*^ mice (7–17 weeks old), while unaltered in the lung, liver, kidney, and pancreas (Fig. 1e, f). Thus, *E*^*BAB*^ is an enhancer specific to thymocytes and splenic T cells (Fig. 1a, b and Supplementary Fig. 1), deletion of which only slightly affected its proximal genes *Bim* and *Bub1* in total thymocytes and splenocytes (Fig. 1e, f).

### *E*^*BAB*^ KO accumulates high-affinity TCR clones in the thymus

**Fig. 2:**
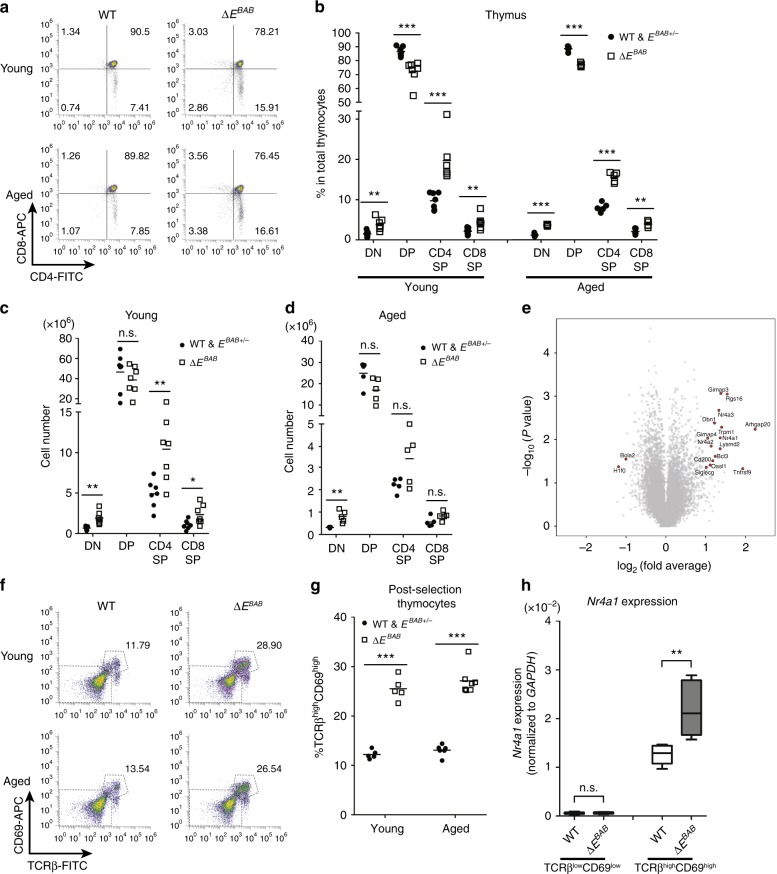
The Δ*E*^*BAB*^ thymus accumulates high affinity TCR clones. **a** Flow cytometric analysis of CD4 versus CD8 thymocyte populations. Data are representative of seven independent experiments (Young; *n* = 7 WT and *E*^*BAB*+/−^–Δ*E*^*BAB*^ littermate pairs, 7–17 weeks old) or five independent experiments (Aged; *n* = 5 WT & *E*^*BAB*+/−^–Δ*E*^*BAB*^ littermate pairs, 30–36 weeks old). **b**–**d** Double negative (DN), double positive (DP), CD4 single positive (SP), and CD8 SP thymocyte proportions of young and aged mice (**b**), and cell numbers of young (**c**) and aged (**d**) mice. Data are pooled from seven independent experiments (Young; *n* = 7 WT and *E*^*BAB*+/−^–Δ*E*^*BAB*^ littermate pairs, 7–17 weeks old) or five independent experiments (Aged; *n* *=* 5 WT and *E*^*BAB*+/-^–Δ*E*^*BAB*^ littermate pairs, 30–36 weeks old). **e** Scatter plot (log_2_ fold change versus –log_10_ *P* value) of genes analyzed by RNA-seq (*n* = 2 WT and *E*^*BAB*+/−^–Δ*E*^*BAB*^ littermate pairs, 10–11 weeks old). Genes showing more than twofold changes with *P* < 0.05 (unpaired two-tailed Student’s *t* test) are highlighted. **f** Flow cytometric analysis of TCRβ versus CD69 thymocyte populations. Data are representative of five independent experiments (Young; *n* = 5 WT and *E*^*BAB*+/−^–Δ*E*^*BAB*^ littermate pairs, 9–12 weeks old, Aged; *n* = 6 WT and *E*^*BAB*+/−^ mice and *n* = 7 Δ*E*^*BAB*^ mice from WT and *E*^*BAB*+/−^–Δ*E*^*BAB*^ littermate pairs and trios, 30–36 weeks old). **g** Post-selection (TCRβ^high^CD69^high^) thymocyte proportion of young and aged mice. Data are pooled from five independent experiments (Young; *n* = 5 WT and *E*^*BAB*+/−^–Δ*E*^*BAB*^ littermate pairs, 9–12 weeks old, Aged; *n* = 6 WT and *E*^*BAB*+/−^ mice, *n* = 7 Δ*E*^*BAB*^ mice, WT and *E*^*BAB*+/−^–Δ*E*^*BAB*^ littermate pair or trio, 30–36 weeks old). **h** qPCR analysis for *Nr4a1* in pre-selection (TCRβ^low^CD69^low^) and post-selection thymocytes of WT and Δ*E*^*BAB*^. Data are pooled from five independent experiments (*n* = 5 sex-matched WT–Δ*E*^*BAB*^ pairs, 10–18 weeks old, mean ± s.d.). Edges of the box are the 25th and 75th percentiles, and error bars extend to the maximum and minimum. Each symbol in (**b**, **c**, **d**, **g**) represents an individual mouse; small horizontal lines indicate the mean. n.s. not significant (*P* ≥ 0.05); *P < 0.05, **P < 0.01, ***P < 0.001 (unpaired two-tailed Student’s *t* test)

Next, we asked whether Δ*E*^*BAB*^ impairs T cell homeostasis in the thymus. For this purpose, thymocytes were analyzed using flow cytometry with anti-CD4 and anti-CD8 antibodies (Fig. 2a–d). While the extent of reduction in *Bim* and *Bub1* expression was very modest in Δ*E*^*BAB*^ thymocytes (Fig. 1e, f), severe abnormalities in thymocyte population were observed both in young (7–17 weeks old) and aged (30–36 weeks old) mice (Fig. 2a, b): Δ*E*^*BAB*^ increased proportion of double negative (DN), CD4 single positive (CD4 SP), and CD8 SP thymocytes, whereas proportion of double positive (DP) had decreased (Fig. 2a, b). These were attributed to the increased number of DN, CD4 SP, and CD8 SP thymocytes (Fig. 2c, d). No obvious phenotypic differences were observed between WT and heterozygotes, between Δ*E*^*BAB*^ males and Δ*E*^*BAB*^ females, and among the three founder lines (Supplementary Fig. 3).

To understand the nature of altered T cell homeostasis in the Δ*E*^*BAB*^ thymus, we performed whole transcriptome analysis of Δ*E*^*BAB*^ thymocytes (two littermate pairs) utilizing RNA-seq (Fig. 2e). As shown in Fig. 2e, only a small number of genes showed statistically significant (*P* < 0.05) more than twofold changes (see methods and Supplementary Data 2 for the detail). Yet, differentially expressed genes (DEGs) affected by Δ*E*^*BAB*^ indicated that the Δ*E*^*BAB*^ thymus accumulated high affinity TCR clones (Fig. 2e): the Δ*E*^*BAB*^ thymus exhibited a higher level of *Nr4a1* expression when compared to the WT thymus. *Nr4a1* is a faithful responder for TCR signal, and the expression level of *Nr4a1* positively correlates with TCR signal strength^15,25,26^. Elevated expression of *Nr4a1* is thus one of the hallmarks of high affinity TCR clones in the thymus. *Arhgap20* and *Tnfrsf9*, the top two most elevated genes in RNA-seq data in the Δ*E*^*BAB*^ thymus (Fig. 2e), are also known as being upregulated in TCR-activated T cells^26^, supporting the notion that high-affinity TCR clones accumulate in Δ*E*^*BAB*^ mice.

To obtain more evidence that the Δ*E*^*BAB*^ thymus piles up high-affinity TCR clones, we stained thymocytes with anti-TCRβ and anti-CD69 antibodies (Fig. 2f, g). We found that proportion of post-selection (TCRβ^high^CD69^high^) thymocytes was increased in the Δ*E*^*BAB*^ thymus both in young and aged mice (Fig. 2f, g). Moreover, as determined by cell-sorting followed by qPCR, *Nr4a1* expression in post-selection thymocytes was much higher in Δ*E*^*BAB*^ than in WT (Fig. 2h). Thus, it was likely that high affinity TCR clones were accumulated in the Δ*E*^*BAB*^ thymus.

We also analyzed TCR^+^ population in the DN subset: thymic precursors of TCRαβ^+^CD8αα^+^ intestinal intraepithelial lymphocytes (IELs)^27,28^ and TCR Vα14^+^ invariant natural killer T cells (iNKT cells)^29,30^. Our data demonstrated that Δ*E*^*BAB*^ increased the number of IEL precursors (Supplementary Fig. 4a, b), while iNKT population was much less affected by Δ*E*^*BAB*^ (Supplementary Fig. 4c–e). These changes may contribute to the increased number of DN thymocytes in Δ*E*^*BAB*^ mice (Fig. 2c, d).

### *E*^*BAB*^ is required for eliminating high-affinity TCR clones

**Fig. 3:**
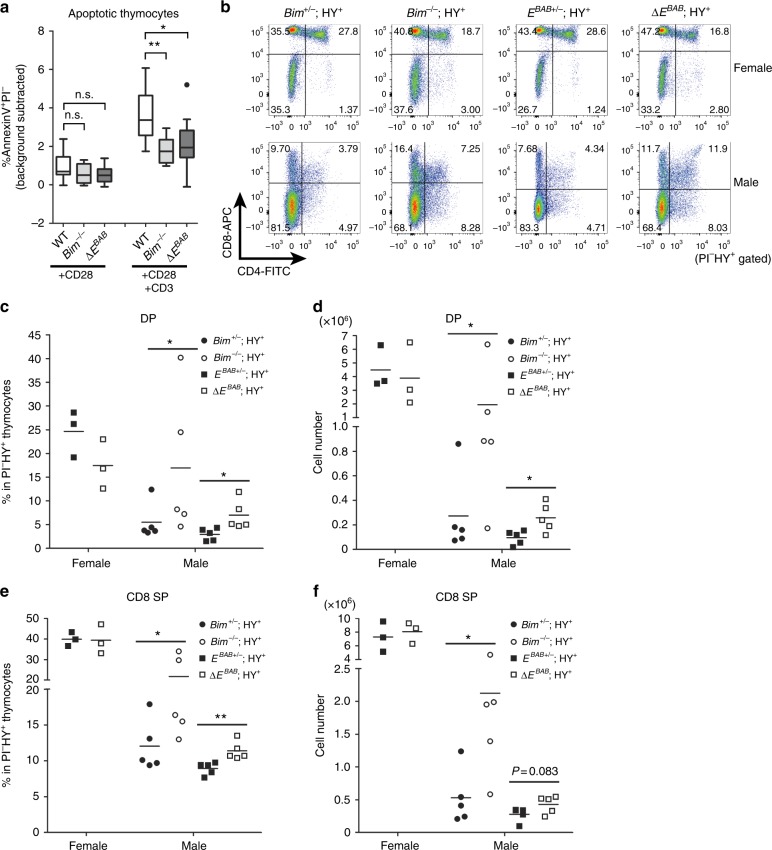
*E*^*BAB*^ contributes to depletion of high affinity TCR clones in ex vivo TCR stimulation and the HY tg models. **a** Annexin V^+^Propidium Iodide (PI)^−^ fraction of total thymocytes stimulated with anti-CD3 and anti-CD28 antibodies (10 µg/ml) for 9 h. The percentage in mock sample (i.e., background) is subtracted. Data are pooled from 11 independent experiments (*n* = 3 WT–Δ*E*^*BAB*^ littermate pairs, *n* = 3 sex-matched WT–Δ*E*^*BAB*^ pairs, *n* = 1 WT–*Bim*^*−/−*^ littermate pair, *n* = 1 sex-matched WT–*Bim*^*−/−*^ pair, *n* = 3 sex-matched WT–*Bim*^*−/−*^–Δ*E*^*BAB*^ trios, 5–20 weeks old). Edges of the box are the 25th and 75th percentiles, and error bars extend to the maximum and minimum. Outliers are defined as the data point that is located outside of *q*_*3*_ + 1.5(*q*_3_ − *q*_1_) and *q*_1_ − 1.5(*q*_3_ − *q*_1_), in which *q*_1_ and *q*_3_ are the 25th and 75th percentiles. **b** CD4 versus CD8 flow cytometric analysis of PI^−^HY-TCR^+^ thymocytes from female and male HY tg mice. The number in the plot is representative percentage of each gate. **c**−**d** DP thymocyte proportion (**c**) and cell numbers (**d**) of PI^−^HY-TCR^+^ thymocytes from female and male HY tg mice. **e**, **f** CD8 SP thymocyte proportion (**e**) and cell numbers (**f**) of PI^−^HY-TCR^+^ thymocytes from female and male HY tg mice. Data are representative of (**b**), or pooled from (**c**–**f**), six independent experiments (*n* = 1 *Bim*^+/−^; HY^+^ and *Bim*^−/−^; HY^+^ female mice, *n* = 5 *Bim*^+/−^; HY^+^ and *Bim*^*−*^^/−^; HY^+^ male mice, *n* = 3 *E*^*BAB*+/−^; HY^+^ and Δ*E*^*BAB*^; HY^+^ female mice, *n* = 5 *E*^*BAB*+/−^; HY^+^ and Δ*E*^*BAB*^; HY^+^ male mice, 6–8 weeks old). Each symbol in (**c**–**f**) represents an individual mouse; small horizontal lines indicate the means. n.s. not significant (*P* ≥ 0.05); *P < 0.05, **P < 0.01 (unpaired one-tailed Student’s *t* test or Mann–Whitney *U* test for *Bim* KO data, and unpaired two-tailed Student’s *t* test for Δ*E*^*BAB*^ data)

Accumulation of high-affinity TCR clones in the Δ*E*^*BAB*^ thymus led us to hypothesize that *E*^*BAB*^ is required for negative selection and/or more general apoptosis processes. To test this hypothesis, we treated primary thymocytes with four apoptotic stimuli: dexamethasone (DEX)^7,8^, phorbol 12-myristate 13-acetate (PMA)^7,8^, ionomycin^7,8^ (Supplementary Fig. 5), and anti-CD3 and anti-CD28 antibodies^6,25^ (Fig. 3a). Δ*E*^*BAB*^ did not affect survival of DEX- or PMA-treated thymocytes (Supplementary Fig. 5a, b) but partially rescued thymocytes from cell death caused by ionomycin or anti-CD3 and anti-CD28 antibodies (Fig. 3a and Supplementary Fig. 5c–e). The rescue in ionomycin experiments was observed in a dose-dependent manner (Supplementary Fig. 5c–e). PMA, ionomycin, and anti-CD3 and anti-CD28 antibodies are often used for artificially activating TCR stimulation and thus for mimicking negative selection ex vivo^6–8,25^. Thus, these results suggest that Δ*E*^*BAB*^ thymocytes were defective in apoptosis following TCR signal activation, leading to a hypothesis that *E*^*BAB*^ plays a role in depleting high-affinity TCR clones.

**Fig. 4:**
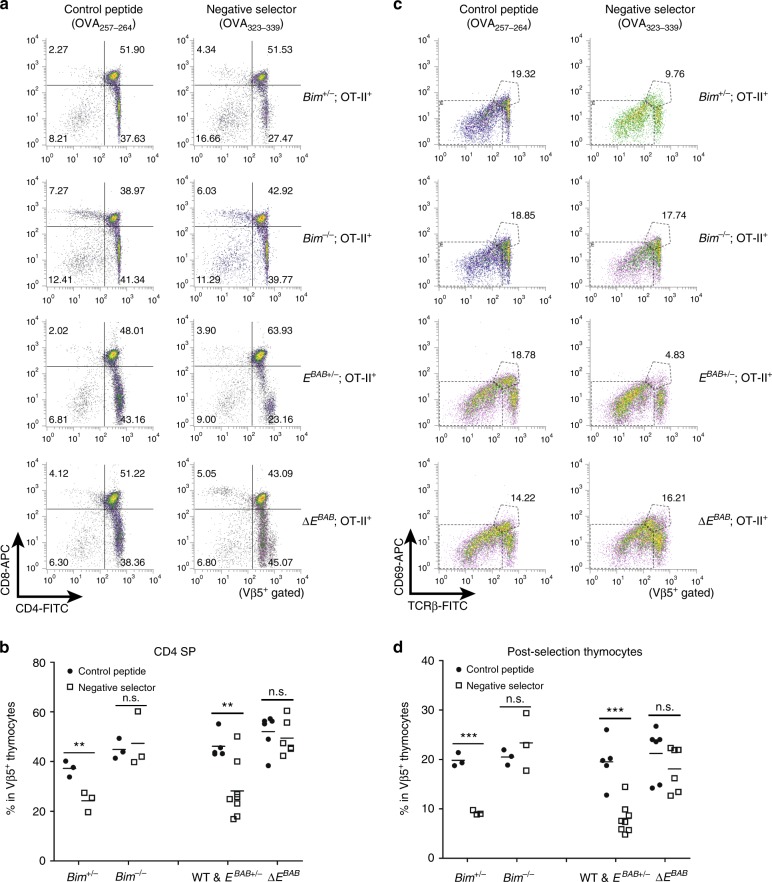
*E*^*BAB*^ is essential for depleting high affinity TCR clones in the OT-II tg model. **a** CD4 versus CD8 flow cytometric analysis of TCR Vβ5^+^ thymocytes from mice injected with OVA_257–264_ or OVA_323–229_ peptide intraperitoneally. The number in the plot is representative percentage of each gate. **b** CD4 SP thymocyte proportion of TCR Vβ5^+^ thymocytes from mice treated as in Fig. 4a. **c** TCRβ versus CD69 flow cytometric analysis of TCR Vβ5^+^ thymocytes from mice treated as in Fig. 4a. The number in the plot is representative percentage of the gate. **d** Post-selection (TCRβ^high^CD69^high^) thymocyte proportion of TCR Vβ5^+^ thymocytes from mice treated as in Fig. 4a. Data are representative of (**a**, **c**) or pooled from (**b**, **d**) 13 independent experiments (*n* = 5 WT and *E*^*BAB*+/−^ mice injected with OVA_257–264_ peptide, *n* = 8 WT and *E*^*BAB*+/−^; OT-II^+^ mice injected with OVA_323–339_ peptide, *n* = 6 Δ*E*^*BAB*^; OT-II^+^ mice injected with OVA_257–264_ and OVA_323–339_ peptide, *n* = 3 *Bim*^+/−^; OT-II^+^ and *Bim*^−/−^; OT-II^+^ mice injected with each peptide, 5–14 weeks old). Each symbol (**b**, **d**) represents an individual mouse; small horizontal lines indicate the mean. n.s. not significant (*P* ≥ 0.05); **P < 0.01, ***P < 0.001 (unpaired one-tailed Student’s *t* test for *Bim* KO data, unpaired two-tailed Student’s *t* test for Δ*E*^*BAB*^ data)

To assess the possibility that *E*^*BAB*^ is required for deleting high affinity TCR clones in vivo, we took advantage of three TCR transgenic mouse models: HY transgenic (tg)^6^ (Fig. 3b–f), OT-II tg^6,31,32^ (Fig. 4), and OT-I tg^33,34,35^ (Supplementary Fig. 6).

HY tg mice express αβTCRs that recognize a male-specific endogenous antigen called HY, which is presented by H-2D^b^ class I MHC molecules^6^ (Fig. 3b-f). Δ*E*^*BAB*^; HY tg mice were analyzed at 6–8 weeks old as described previously^6^ by flow cytometry. As shown by the lower abundance of DP and CD8 SP in *E*^*BAB*+/−^; HY tg male mice compared to female mice (Fig. 3b–f), thymocytes expressing HY-TCR are negatively selected in a male-specific manner. Notably, Δ*E*^*BAB*^ significantly rescued DP (both in proportion and number) and CD8 SP (in proportion) thymocytes in HY tg male mice (Fig. 3b–f).

OT-II tg thymocytes express Vα2/Vβ5 TCRs that primarily recognize chicken ovalbumin-derived peptide (ISQAVHAAHAEINEAGR, OVA_323–339_ peptide) presented by I^-^A^b^ class II MHC molecules^6,31,32^. Δ*E*^*BAB*^; OT-II tg mice were generated by crossing and injected with OVA_323–339_ peptide or the control peptide intraperitoneally. WT; OT-II tg or *E*^*BAB*+/−^; OT-II tg mice were also used as a control. In response to injection with OVA_323–339_ peptide, the CD4 SP proportion of control thymocytes was massively reduced (Fig. 4a, b). Strikingly, *E*^*BAB*^ KO rescued this reduction (Fig. 4a, b). Staining thymocytes with anti-TCRβ and anti-CD69 antibodies revealed that Δ*E*^*BAB*^ almost completely prevented the deletion of post-selection thymocytes caused by OVA_323–339_ peptide injection (Fig. 4c, d).

Fetal thymic organ culture (FTOC) with the OT-I tg system allows us to investigate effects of antigens of interests on intrathymic T cell development^33–36^. OT-I tg thymocytes express Vα2/Vβ5 TCRs that bind chicken ovalbumin peptide residues 257–264 (SIINFEKL, OVA_257–264_ peptide) in the context of H-2K^b^ class I MHC molecules^34^. Of note, a series of OVA_257–264_ variants with different TCR affinity can be used in the OT-I tg FTOC system^34,35^. We cultured fetal thymus (FT) of *E*^*BAB*+/−^; OT-I tg and Δ*E*^*BAB*^; OT-I tg in the presence of OVA_257–264_, Q4R7 (SIIQFERL), or gp33 (KAVYNFATC)^34^ (Supplementary Fig. 6). The previous publications establish OVA_257–264_ and Q4R7 as negative selectors (affinity to OT-I TCR: OVA_257–264_ > Q4R7)^34,35^. gp33, which does not bind to OT-I TCRs, was used as a control. Δ*E*^*BAB*^ compared to *E*^*BAB+/−*^ significantly rescued DP thymocytes from Q4R7-dependent selection but not those from OVA_257–264_-dependent selection (Supplementary Fig. 6). Thus, Δ*E*^*BAB*^ rescued OT-I TCR^+^ thymocytes from negative selection in a TCR affinity-dependent manner.

Taken the results from the three transgenic models together, we concluded that *E*^*BAB*^ plays an important role in apoptosis of high-affinity TCR clones in thymic negative selection ex vivo and in vivo.

### *E*^*BAB*^ is essential for TCR-dependent activation of *Bim*

**Fig. 5:**
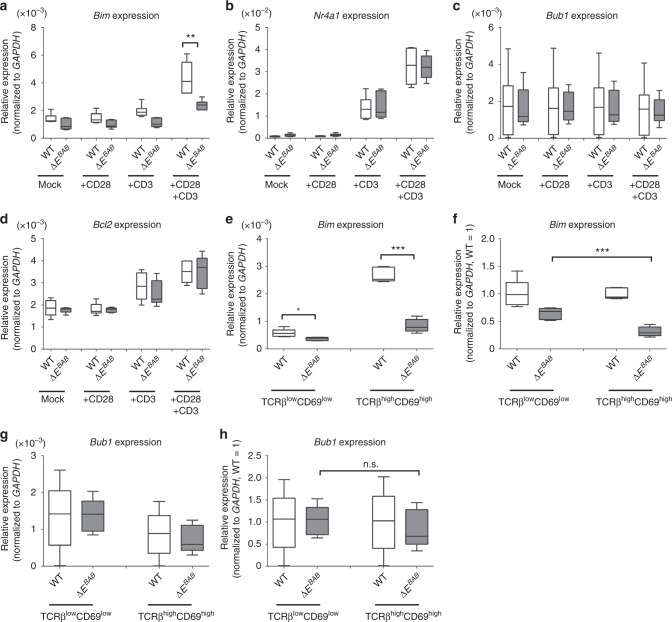
*E*^*BAB*^ regulates *Bim* expression upon TCR stimulation. **a**–**d** Gene expression changes for *Bim* (**a**), *Nr4a1* (**b**), *Bub1* (**c**), and *Bcl2* (**d**) in total thymocytes stimulated for 3 h with anti-CD3 and anti-CD28 antibodies (10 µg/ml). **e**, **f** Gene expression level (**e**) and relative gene expression to WT (**f**) for *Bim* in pre-selection (TCRβ^low^CD69^low^) and post-selection (TCRβ^high^CD69^high^) thymocytes. **g**, **h** Gene expression level (**g**) and relative gene expression to WT (**h**) for *Bub1* in pre-selection (TCRβ^low^CD69^low^) and post-selection (TCRβ^high^CD69^high^) thymocytes. Data are pooled from six independent experiments (**a**–**d**; *n* = 6 sex-matched WT–Δ*E*^*BAB*^ pairs, 9–18 weeks old) or five independent experiments (**e**–**h**; *n* = 5 sex-matched WT–Δ*E*^*BAB*^ pairs, 10–18 weeks old). Edges of the box are the 25th and 75th percentiles, and error bars extend to the maximum and minimum. Outliers are defined as the data point that is located outside of *q*_*3*_ + 1.5(*q*_3_ − *q*_1_) and *q*_1_ − 1.5(*q3* − *q*_1_), in which *q*_1_ and *q*_3_ are the 25th and 75th percentiles. n.s. not significant (*P* ≥ 0.05); *P < 0.05, **P < 0.01, ***P < 0.001 (unpaired two-tailed Student’s *t* test)

The above-described phenotypes observed in the Δ*E*^*BAB*^ thymus, four ex vivo culture systems, and three transgenic models are strikingly similar to those observed in *Bim* KO mice as described in multiple publications^6–10^ and as validated by ourselves (Figs. 2–4 and Supplementary Figs. 4–7). For example, the extent of increase for post-selection thymocytes caused by Δ*E*^*BAB*^ was similar to that by *Bim* KO (Fig. 2g. versus Supplementary Fig. 7e). Such similarity was also observed for the ex vivo TCR stimulation (Fig. 3a), OT-II (Fig. 4), and OT-I (Supplementary Fig. 6) experiments, whereas *Bim* KO seemingly more efficiently rescued HY-TCR^+^ thymocytes than *E*^*BAB*^ KO did in the HY experiments (Fig. 3: see discussion regarding interpretation on this data). Moreover, Δ*E*^*BAB*^ did not affect DEX-induced cell death, which was in fact *Bim*-independent (Supplementary Fig. 5a). Some of the thymocytes abnormalities are seen also in mice ectopically expressing Bim-antagonist Bcl2 (Bcl2 tg) in a T cell-specific manner, which leads to defective apoptosis in the thymus^25,37,38^. Although Δ*E*^*BAB*^ had a minor impact on *Bim* expression in total thymocytes (Fig. 1e), we next examined whether the deficient negative selection of Δ*E*^*BAB*^ thymocytes is due to abnormal regulation of *Bim*. To this end, we again artificially activated TCR by treating thymocytes with anti-CD3 and anti-CD28 antibodies ex vivo, and then measured expression of *Bim*, *Nr4a1*, *Bub1*, and *Bcl2* by qPCR (Fig. 5a–d). *Bim* expression in Δ*E*^*BAB*^ thymocytes treated with anti-CD3 and anti-CD28 antibodies was clearly lower than that in TCR-stimulated WT thymocytes (Fig. 5a). Expression of *Nr4a1*, a faithful responder for TCR signal, was comparable between WT and Δ*E*^*BAB*^ (Fig. 5b), suggesting that TCR signal was properly activated in Δ*E*^*BAB*^ thymocytes. Consistent with the report that a dominant negative form of *Bub1* is insufficient to cause T cell abnormalities^39^, *Bub1* expression was not influenced by TCR stimulation and *E*^*BAB*^ KO (Fig. 5c). *Bcl2*, a TCR-downstream anti-apoptotic gene, exhibited an expected response to TCR stimulation^40^, and this response was comparable between WT and Δ*E*^*BAB*^ (Fig. 5d). Thus, it was likely that *E*^*BAB*^ primarily targets *Bim* following TCR signal.

To assess the role of *E*^*BAB*^ in TCR-dependent *Bim* activation in vivo, we sorted pre- and post-selection thymocytes and measured expression of *Bim* and *Bub1* (Fig. 5e–h). If *E*^*BAB*^ regulates *Bim* in a TCR signal dependent manner, *Bim* expression should be affected by Δ*E*^*BAB*^ at the post-selection stage while less affected at the pre-selection stage. As expected, expression of *Bim* in the post-selection (TCRβ^high^CD69^high^) thymocytes was markedly lower in Δ*E*^*BAB*^ than in WT (Δ*E*^*BAB*^/WT = 0.31) (Fig. 5e, f). Although *Bim* expression was moderately affected by *E*^*BAB*^ KO in the pre-selection (TCRβ^low^CD69^low^) stage (Δ*E*^*BAB*^/WT = 0.64) (Fig. 5e, f), expression ratio of Δ*E*^*BAB*^ to WT in the post-selection stage was significantly lower than that in the pre-selection stage (Fig. 5f). These results were validated at the protein level, demonstrating that protein expression of BimEL and BimL, two of major isoforms of Bim^41^, was compromised by Δ*E*^*BAB*^ (Supplementary Fig. 9). Together, Δ*E*^*BAB*^ affected *Bim* expression in a post-selection-biased manner, indicating an important role of *E*^*BAB*^ in activating *Bim* upon TCR activation in vivo. *Bub1* expression was not significantly altered between Δ*E*^*BAB*^ and WT mice both in pre- and post-selection thymocytes (Fig. 5g, h), again excluding *Bub1* in explaining the T cell phenotypes we observed (Figs. 2–4). Collectively, our data demonstrated that *E*^*BAB*^ KO disrupts transcriptional activation for *Bim* upon TCR stimulation, resulting in the rescue of high affinity TCR clones in the thymus (Figs. 2–5). We reasoned that *Bim* expression in total thymocytes looked only mildly affected by *E*^*BAB*^ KO (Fig. 1e) as only 10–30% of thymocytes is at the post-selection stage where transcriptional regulation of *Bim* is strongly affected by Δ*E*^*BAB*^.

Furthermore, we investigated whether *E*^*BAB*^ regulates *Bim* in *cis* or *trans*. To test this, we generated *E*^*BAB*+/−^; *Bim*^−/+^ mice (Supplementary Fig. 10a). If *E*^*BAB*^ controls *Bim* in *cis*, a phenotype of *E*^*BAB*+/−^; *Bim*^−/+^ thymocytes should be similar to Δ*E*^*BAB*^ (Supplementary Fig. 10a). If *E*^*BAB*^ can regulate *Bim* in *trans*, *E*^*BAB*+/−^; *Bim*^−/+^ mice should show no phenotype in the thymus (Supplementary Fig. 10a). Staining with anti-CD4, anti-CD8, anti-TCRβ, and anti-CD69 antibodies revealed that *E*^*BAB*+/^^−^; *Bim*^−^^/+^ thymocytes exhibited Δ*E*^*BAB*^-like phenotypes (Supplementary Fig. 10b–f). Given that *Bim*^+/−^ exhibits almost no phenotype in DN, DP, CD4 SP, CD8 SP, and TCRβ^high^CD69^high^ thymocytes (Supplementary Fig. 10g), these results validated that *E*^*BAB*^ is a *cis*-regulatory element regulating *Bim*.

### *E*^*BAB*^ is dispensable for T_reg_ cells and peripheral T cells

**Fig. 6:**
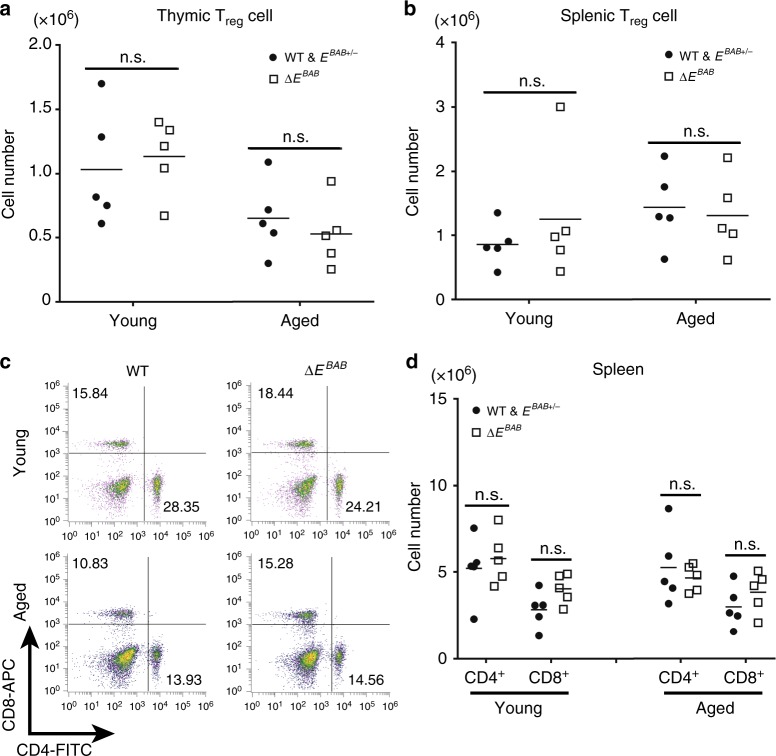
*E*^*BAB*^ does not play a major role in maintaining homeostasis of regulatory T cells and peripheral T cells. **a**, **b** CD4^+^Foxp3^+^ thymic (**a**) and splenic (**b**) T_reg_ cell numbers of young and aged mice. Data are pooled from five independent experiments (Young; *n* = 5 WT and *E*^*BAB*+/−^–Δ*E*^*BAB*^ littermate pairs, 9–12 weeks old, Aged; *n* = 5 WT *E*^*BAB*+/−^–Δ*E*^*BAB*^ littermate pairs, 30–36 weeks old). **c** Flow cytometric analysis of CD4 versus CD8 T cell populations in the spleen. The number in the plot is representative percentage of each gate. Data are representative of five independent experiments (Young; *n* = 5 WT and *E*^*BAB*+/−^–Δ*E*^*BAB*^ littermate pairs, 7–17 weeks old, Aged; *n* *=* 5 WT and *E*^*BAB*+/−^–Δ*E*^*BAB*^ littermate pairs, 30–36 weeks old). **d** CD4^+^ and CD8^+^ splenic T cell numbers. Data are pooled from five independent experiments (Young; *n* = 5 WT and *E*^*BAB*+/−^–Δ*E*^*BAB*^ littermate pairs, 7–17 weeks old, Aged; *n* *=* 5 WT and *E*^*BAB*+/−^–Δ*E*^*BAB*^ littermate pairs, 30–36 weeks old). Each symbol in (**a**, **b**, **d**) represents an individual mouse; small horizontal lines indicate the mean. n.s. not significant (*P* ≥ 0.05) (unpaired two-tailed Student’s *t* test)

Expression levels of Nr4a transcription factors including Nr4a1 are markers not only for high affinity TCR clones, but also for thymic T_reg_ cells that suppress autoreactive T cells in periphery^1,5^. Nr4a transcription factors cooperatively promote T_reg_ differentiation through directly activating expression of *Foxp3*, the master regulator for T_reg_ identity^42–44^. Thus, it is thought that high-affinity TCR clones are a precursor of T_reg_ cells^5^. *Bim* KO and Bcl2 tg mice accumulate both of high affinity TCR clones and T_reg_ cells^6–8,14,15,25,37,38^ (Supplementary Figs. 7d, e, 8c). To examine whether accumulation of high-affinity TCR clones resulted in the increased number of T_reg_ cells in Δ*E*^*BAB*^ mice, we analyzed thymic and splenic T_reg_ cells by staining with anti-Foxp3 antibody (Fig. 6a, b). We found that the number of thymic and splenic T_reg_ cells were comparable between WT and Δ*E*^*BAB*^ (Fig. 6a, b). Thus, although high-affinity TCR clones are accumulated in the Δ*E*^*BAB*^ thymus (Fig. 2), *E*^*BAB*^ does not affect homeostasis of T_reg_ cells. These results indicate that *Bim*-dependent homeostasis of T_reg_ cells in the thymus and spleen is independent on *E*^*BAB*^.

*Bim* KO and Bcl2 tg mice also accumulate peripheral T cells^6–8,12,13,25,37,38^ (Supplementary Fig. 8a, b). However, the number of splenic CD4^+^ and CD8^+^ T cell were not affected by Δ*E*^*BAB*^ both in young and aged mice (Fig. 6c, d). In addition, splenic B cells, whose homeostasis depends on *Bim*^8^, were unaffected by *E*^*BAB*^ KO (Supplementary Fig. 11). These results indicated that homeostasis of peripheral T cells is properly maintained in Δ*E*^*BAB*^ mice in contrast to *Bim* KO mice^6–8,12,13^ (Supplementary Fig. 8a, b).

**Fig. 7:**
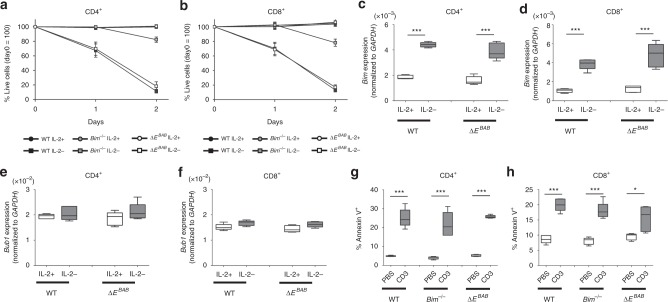
*E*^*BAB*^ is dispensable for two apoptotic pathways in peripheral T cells. **a**, **b** Viability (% Annexin V^−^PI^−^) of activated CD4^+^ (**a**) and CD8^+^ (**b**) splenic T cells after IL-2 withdrawal. Data are pooled from ten independent experiments (**a**; *n* = 5 sex-matched WT–Δ*E*^*BAB*^ pairs and *n* = 5 sex-matched WT–*Bim*^−*/−*^–Δ*E*^*BAB*^ trios, 5–26 weeks old, mean ± s.d.) or 11 independent experiments (**b**; *n* = 5 sex-matched WT–Δ*E*^*BAB*^ pairs and *n* = 5 sex-matched WT–*Bim*^*−/−*^–Δ*E*^*BAB*^ trios, 5–26 weeks old, mean ± s.d.). **c**, **d** Gene expression level for *Bim* in activated CD4^+^ (**c**) and CD8^+^ (**d**) splenic T cells cultured with or without IL-2 for 6 h. Data are pooled from five independent experiments (*n* = 5 sex-matched WT–*Bim*^−*/−*^–Δ*E*^*BAB*^ trios, 5–14 weeks old). **e,** **f** Expression of *Bub1* in activated CD4^+^ (**e**) and CD8^+^ (**f**) splenic T cells cultured with or without IL-2 for 6 h. Data are pooled from five independent experiments (*n* = 5 sex-matched WT–*Bim*^*−/−*^–Δ*E*^*BAB*^ trios, 5–14 weeks old). **g**, **h** Apoptotic cell rate (%Annexin V^+^) of activated CD4^+^ (**g**) and CD8^+^ (**h**) splenic T cells restimulated with anti-CD3 antibody (5 µg/ml) for 6 h. Data are pooled from five independent experiments (*n* = 5 sex-matched WT–*Bim*^*−/−*^–Δ*E*^*BAB*^ trios, 5–26 weeks old). Edges of the box are the 25th and 75th percentiles, and error bars extend to the maximum and minimum. Outliers are defined as the data point that is located outside of *q*_3_ + 1.5(*q*3 − *q*_1_) and *q*_1_ − 1.5(*q*_3_ − *q*_1_), in which *q*_1_ and *q*_3_ are the 25th and 75th percentiles (**c**–**h**). n.s. not significant (*P* ≥ 0.05); *P < 0.05, **P < 0.01, ***P < 0.001 (unpaired two-tailed Student’s *t* test)

To gain an additional insight into a role of *E*^*BAB*^ in peripheral T cell homeostasis, we performed Interleukin-2 (IL-2) deprivation experiments and activation-induced cell death (AICD) experiments in cultured activated CD4^+^ and CD8^+^ splenocytes (Fig. 7).

*Bim*-deficient peripheral T cells are resistant to the absence of IL-2 ex vivo^7,45^ while WT and Δ*E*^*BAB*^ splenic T cells needed IL-2 for their continuous survival in culture (Fig. 7a, b). In accordance with this, *E*^*BAB*^ was dispensable for upregulation of *Bim* following IL-2 withdrawal (Fig. 7c, d). Expression of *Bub1* was merely affected by IL-2 (Fig. 7e, f). Hence, IL-2 deprivation-dependent T cell death in periphery requires *Bim* but not *E*^*BAB*^.

AICD is known as a peripheral cell death cascade where repeatedly activated T cells undergo apoptosis^46^. In fact, reactivating peripheral T cells by anti-CD3 antibody treatment strongly induced cell death (Fig. 7g, h). Using this system, we found that neither *Bim* nor *E*^*BAB*^ was required for AICD ex vivo (Fig. 7g, h).

From these experiments, we concluded that at least two peripheral apoptotic pathways are intact in Δ*E*^*BAB*^ mice, which can be one explanation for why the increase in thymic high-affinity TCR clones did not lead to accumulation of peripheral T cells.

**Fig. 8:**
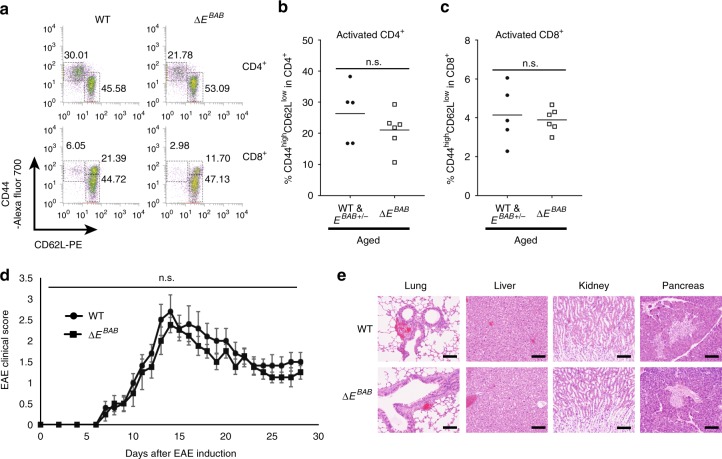
Δ*E*^*BAB*^ mice do not show any autoimmune phenotype. **a** Flow cytometric analysis of CD62L versus CD44 populations in CD4^+^ or CD8^+^ splenic T cell. The number in the plot is representative percentage of each gate. For CD4^+^ T cells, gates indicate activated (CD44^high^CD62L^low^) and naïve (CD44^low^CD62L^high^) populations. For CD8^+^ T cells, activated effector memory (CD44^high^CD62L^low^), activated central memory (CD44^high^CD62L^high^) and naïve (CD44^low^CD62L^high^) populations are gated. Data are representative of four independent experiments (*n* = 5 for WT and *E*^*BAB*+/−^ mice, *n* = 6 for Δ*E*^*BAB*^ mice from WT & *E*^*BAB*+/−^–Δ*E*^*BAB*^ littermate pairs and trios, 30–34 weeks old). **b**, **c** Activated CD4^+^ (**b**) and CD8^+^ (**c**) proportions of aged mice. Data are pooled from four independent experiments (*n* = 5 for WT and *E*^*BAB*+/−^ mice, *n* = 6 for Δ*E*^*BAB*^ mice from WT and *E*^*BAB*+/−^–Δ*E*^*BAB*^ littermate pairs and trios, 30–34 weeks old). **d** The mean (±s.e.m.) clinical scores at the days after EAE was induced in WT (control) (*n* = 10) and Δ*E*^*BAB*^ mice (*n* = 8). The incidence of EAE: control 10/10, Δ*E*^*BAB*^ 8/8. No data point showed statistically significant difference between WT and Δ*E*^*BAB*^ (i.e., unpaired two-tailed Student’s *t* test *P* ≥ 0.05). **e** Representative pictures for Hematoxylin and Eosin staining for the lung, liver, kidney, and pancreas of two independent experiments (*n* = 2 WT and *E*^*BAB*+/−^–Δ*E*^*BAB*^ littermate pairs, 30–31 weeks old). The scale bars represent 100 μm. Each symbol in (**b**, **c**) represents an individual mouse; small horizontal lines indicate the mean. n.s. not significant (*P* ≥ 0.05) (unpaired two-tailed Student’s *t* test)

We additionally analyzed splenic activated T cell proportion by staining splenocytes with anti-CD44 and anti-CD62L antibodies to investigate autoimmune pathology (Fig. 8a–c). In line with the fact that two peripheral apoptosis pathways are intact in Δ*E*^*BAB*^ mice (Fig. 7), activated T cell (CD44^high^CD62L^low^) proportion was not increased in the Δ*E*^*BAB*^ spleen even in aged mice (Fig. 8a–c and Supplementary Fig. 12a–e). These data were in line with RNA-seq data from WT and Δ*E*^*BAB*^ spleen (two littermate pairs), where only two candidate DEGs were identified (Supplementary Fig. 12f and Supplementary Data 3). In contrast, *Bim*-deficient mice accumulated activated CD8^+^ T cells in the spleen already at younger age (Supplementary Fig. 8d–f).

Furthermore, we assessed a role of *E*^*BAB*^ in an in vivo autoimmune disease model, experimental autoimmune encephalomyelitis (EAE)^47^. As shown in Fig. 8d, clinical scores of Δ*E*^*BAB*^ were comparable to those of WT: Δ*E*^*BAB*^ mice neither exhibited severer nor ameliorated EAE phenotypes, suggesting that *E*^*BAB*^ is dispensable for EAE. In contrast, we confirmed previous publication that *Bim* plays a role in EAE (Supplementary Fig. 13)^16^. Consistently, we did not find any histological sign for autoimmunity such as massive infiltration of leukocytes (i.e., inflammation) in several nonlymphoid organs in Δ*E*^*BAB*^ mice (Fig. 8e).

**Fig. 9:**
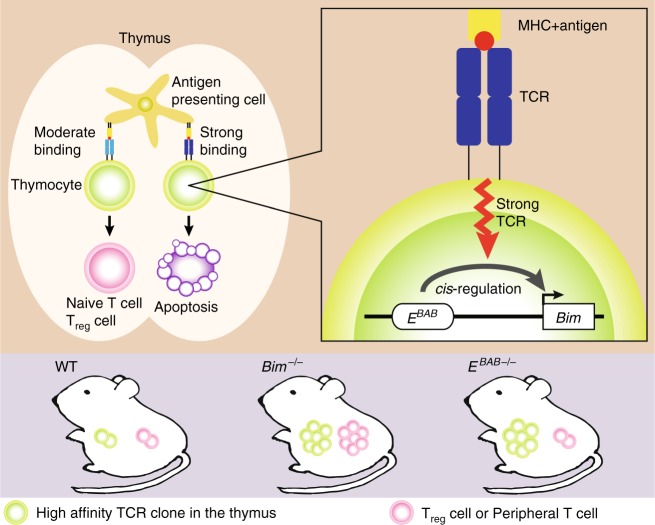
Graphical summary. *E*^*BAB*^ plays a role in inducing TCR signal-dependent apoptosis to eliminate high affinity TCR clones in the thymus while dispensable for *Bim*-dependent peripheral T cell tolerance

Thus, Δ*E*^*BAB*^ mice did not show any autoimmune phenotype, which was consistent with that *E*^*BAB*^ was not essential for peripheral T cell homeostasis (Figs. 6–8, Supplementary Fig. 8, and Supplementary Figs. 12 and 13). These results highlighted an extraordinary specialized role of *E*^*BAB*^ in thymic negative selection (Fig. 9).

## Discussion

It is thought that TCR signal activates *Bim* to promote apoptosis in thymic negative selection process^1–3^. However, it has been unclear how TCR signal strength is linked to the *Bim* expression level. Even whether activation of *Bim* expression is critical for negative selection has been unanswered due to lack of a mutant specifically deficient for TCR-dependent *Bim* expression^17^. Here, by means of CRISPR–Cas9-mediated KO approach in mice, we generated a mutant that lacks a *cis*-regulatory enhancer for *Bim*, *E*^*BAB*^ (Figs. 1–5 and Supplementary Fig. 10). *E*^*BAB*^ is T cell-specific and evolutionarily conserved (Fig. 1a, b and Supplementary Fig. 1), and Δ*E*^*BAB*^ mice were unable to efficiently eliminate high affinity TCR clones (Figs. 2–4 and Supplementary Fig. 6). This defect appeared to be attributed to insufficient activation of *Bim* during TCR activation (Fig. 5 and Supplementary Fig. 9). Thus, we propose that *E*^*BAB*^ links TCR signal and *Bim* expression, contributing to depletion of high affinity TCR clones in the thymus and thus to central T cell tolerance (Fig. 9).

*Bim* KO affects homeostasis of nearly all of T cell types^6–18^ (Supplementary Fig. 4 and Supplementary Figs. 7 and 8). Comparing T cell phenotypes between *Bim* KO and Δ*E*^*BAB*^ mice provides insights into the extent of contribution of *E*^*BAB*^-dependent *Bim* regulation on phenotypes of interests. Among the phenotypes observed in the Δ*E*^*BAB*^ thymus, accumulation of post-selection thymocytes in the Δ*E*^*BAB*^ thymus was as severe as that in the *Bim* KO thymus (Fig. 2f, g and Supplementary Fig. 7d, e). Moreover, both of Δ*E*^*BAB*^ and *Bim* KO rescued thymocytes from negative selection in the ex vivo TCR stimulation model and three transgenic systems (Figs. 3 and 4 and Supplementary Fig. 6). The degrees of rescues by *E*^*BAB*^ KO and *Bim* KO were similar in the ex vivo TCR stimulation, OT-I, and OT-II experiments (Figs. 3 and 4 and Supplementary Fig. 6). In contrast, in the HY experiments, it appeared that *Bim* KO better rescued HY-TCR^+^ thymocytes than *E*^*BAB*^ KO. When interpreting these results, it should be noted that readouts used (e.g., proportion of cell types of interests) do not always specifically reflect negative selection. In particular, *Bim* KO is able to rescue apoptosis induced by various stimuli^18^, even death of control-treated cells (Supplementary Fig. 5). Thus, increased survival seen in *Bim* KO potentially reflects both negative selection-dependent and -independent events such as post-selection lifetime. Importantly, our analyses showed that function of *E*^*BAB*^ appears to be specific for TCR signal-dependent *Bim* expression in the thymus. Hence, we assume that the more efficient rescue observed in *Bim* KO in the HY experiments may be attributed to pleiotropic effects of *Bim* KO on negative selection-independent phenomena in the thymus. Alternatively, the observations in the HY experiments imply that there may be another enhancer controlling TCR-dependent expression of *Bim*. Collectively, we suggest that a role for *Bim* in eliminating high affinity TCR clones in the thymus requires *E*^*BAB*^. These results establish *E*^*BAB*^-mediated transcriptional regulation for *Bim* as a contributor for *Bim*-dependent thymic negative selection.

*Bim* KO and Bcl2 tg mice accumulate T_reg_ cells, a basis for the current model that T_reg_ cells are differentiated from high-affinity TCR clones in the thymus^1,5,15,25^ (Supplementary 8c). Intriguingly, in the Δ*E*^*BAB*^ thymus, the number of T_reg_ cells was comparable with that in the littermate controls even though high affinity TCR clones were increased (Fig. 2f, g and Fig. 6a, b). This indicates that the residual amount of *Bim* in the Δ*E*^*BAB*^ thymus is sufficient for maintaining T_reg_ homeostasis. It is also plausible that a non-cell autonomous mechanism may be dominant for T_reg_ homeostasis in the thymus. Supporting this, a previous study shows that Foxp3 overexpression induces Bim phosphorylation and enhances apoptosis in newly arising thymic T_reg_ cells^48^. In addition, T_reg_ homeostasis is controlled by availability of pro-survival cytokines such as IL-2 derived from the surrounding environment^48,49^. These altogether indicate that T_reg_ apoptosis is precisely tuned by multiple mechanisms independent from *E*^*BAB*^, and demonstrate that the role of *E*^*BAB*^ is specific for thymic negative selection (Fig. 9). Alternatively, the increased proportion of IEL precursors both in *Bim*^*−/−*^ and Δ*E*^*BAB*^ mice (Supplementary Fig. 4a, b) suggests that *E*^*BAB*^ controls fate determination of survived high-affinity TCR clones: Δ*E*^*BAB*^ may bias fate of thymocytes escaped from negative selection toward IEL rather than T_reg_ cells.

*Bim* KO and Bcl2 tg mice show abnormalities not only in thymic negative selection, but also in peripheral T cell homeostasis^6–8,11–13,25,37,38^ (Supplementary Fig. 8a, b and Supplementary Fig. 12). In contrast, peripheral T cells did not accumulate in the Δ*E*^*BAB*^ spleen (Fig. 6c, d). Consistent with this, Δ*E*^*BAB*^ affects neither peripheral T cell activation nor experimental autoimmunity (Fig. 8 and Supplementary Fig. 12). This suggests that high-affinity TCR clones were peripherally depleted in Δ*E*^*BAB*^ mice. One possible explanation for this observation is that peripherally activated T cells properly undergo IL-2 withdrawal-dependent apoptosis, a process that requires *Bim* but not *E*^*BAB*^ (Fig. 7a, b). This idea is further supported by that *Bim* was properly upregulated upon IL-2 withdrawal in activated T cells even in the absence of *E*^*BAB*^ (Fig. 7c, d). Additional peripheral T cell-specific enhancer(s) for *Bim*, or completely different mechanisms such as post-translational modifications, may also ensure *Bim*-mediated maintenance for peripheral T cell homeostasis. A different apoptosis pathway such as the Fas pathway could also be a major contributor in eliminating peripheral autoreactive T cells^1,46,50,51^. The Fas pathway is known to be required for AICD, which we found requires neither *Bim* nor *E*^*BAB*^ (Fig. 7g, h). These results are consistent with that deletion of *E*^*BAB*^ had a very minor impact on genome-wide gene expression in peripheral T cells (Supplementary Fig. 12f). Collectively, our findings emphasize that the role of *E*^*BAB*^ is dedicated to thymic negative selection (Fig. 9).

A physiological contribution of a gene in a certain biological phenomenon depends on quantity of functional gene products that exist in that context. Regarding classical protein-coding genes, concentration of functional gene products-(modified) proteins is affected by transcriptional regulation, mRNA stability, translational efficacy, post-translational modifications, and protein stability, which are interconnected with each other. Each of these mechanisms profoundly contributes to determine the steady-state concentration of gene products, depending on genes and biological contexts. However, it has been technically difficult to dissect the relative importance of each mechanism for a gene to exert its physiological role(s) in a certain biological situation. Our study is unique in that we disrupted the single gene expression program by targeting the specific *cis*-element. This enabled us to reveal the degrees of contribution of *Bim* and *E*^*BAB*^ in thymic negative selection measured by different readouts, which represent cutting-edge molecular dissection of the *cis*-regulatory control for thymic negative selection in vivo. Although we do not exclude a possibility that downstream mechanisms such as Bim phosphorylation might be also important, we provide the evidence that the enhancer-driven gene expression regulation of *Bim* is an important upstream mechanism in thymic negative selection. In addition, the present work provides an example for using enhancer KO approach, instead of conventional genetic-manipulation of protein-coding genes, to address important biological questions.

## Methods

### Mice

All animal protocols were approved by the Animal Care and Use committee of Advanced Telecommunications Research Institute International (permission numbers: AN20140002, AN20150002, AN20160002, AN20170002, and AN20180002) and Kyoto university (permission numbers: K-16-12-11 and L-18-1). For generating Δ*E*^*BAB*^ mice, four gRNAs were individually cloned into the pX330 vector (#42230, Addgene, MA, USA) that harbors a *Cas9* transgene. Briefly, two pairs of oligos (Supplementary Data 1) were annealed at 95 °C followed by natural cooling to 25 °C, and ligated with BbsI (New England Biolabs, MA, USA)-digested pX330 by using Ligation high (TOYOBO, Osaka, Japan). The obtained plasmids were sequence-validated (FASMAC, Kanagawa, Japan). The four gRNAs were together injected into fertilized eggs of C57BL/6N mice at the concentration of 2.5 μg/μl each (Transgenic, Fukuoka, Japan), generating the three different founders #44, #47, and #50. These founders were crossed with WT to obtain F1. Deleted regions were validated by sequencing analyses (FASMAC) against DNA from F0 and F1. The obtained F1 offspring were then crossed to generate Δ*E*^*BAB*^ mice. *Bim*^−/−^ (B6.129S1-*Bcl2l11*^*tm1.1Ast*^/J) and OT-II tg (B6.Cg-Tg(TcrαTcrβ)425Cbn/J) mice were purchased from The Jackson Laboratory (ME, USA). OT-II tg, OT-I tg and HY tg mice were crossed with Δ*E*^*BAB*^ and *Bim*^*−/*^^−^ mice for negative selection assays.

In all mice experiments, KO and the littermate control (WT or heterozygote), thus in total two to three mice were sacrificed and analyzed side-by-side on the same day to be considered as a littermate pair or trio. All experiments were performed without randomization. Blinding was performed in the injection process of OT-II negative selection assay. In other experiments, blinding was not done.

### DNA isolation and genomic PCR

Genomic DNAs were prepared by using MightyAMP DNA polymerase kit (TaKaRa, Shiga, Japan) according to the manufacturer’s instruction. Genomic PCR experiments were performed using MightyAMP DNA polymerase or KOD FX-neo (TOYOBO) and the primers listed in Supplementary Data 1.

### Epigeome analysis

Epigenome analyses were performed with the UCSC genome browser (https://genome.ucsc.edu/). For analyzing epigenome from peripheral naïve T cells, ChIP-seq data retrieved (accession numbers: GSE67443 and GSE60005) were mapped to the mm9 of mouse genome by using Bowtie2^52^. The obtained sam format files were converted into bam format files by using samtools^53^ that were then subjected to peak calling with MACS^54^. Wig format files were then uploaded into the UCSC genome browser to be visualized.

### RNA isolation, cDNA synthesis, and qPCR

For nonlymphoid organs, total RNAs were prepared by using Trizol reagent (Thermo Fisher Scientific, MA, USA). The obtained supernatants containing total RNAs were further purified with RNeasy mini kit (Qiagen, Venlo, Netherlands) according to the manufacturer’s instruction. Total RNAs from lymphocyte samples were prepared by RNeasy mini kit with the equipped lysis buffer. 0.1–2 μg of total RNAs were then reverse transcribed with SuperScript III first-strand synthesis system (Thermo Fisher Scientific) according to the manufacturer’s instruction. The obtained cDNAs were 5- or tenfold-diluted and subjected into qPCR experiments by using LightCycler480 Instrument II system and SYBR Green Master Mix (Roche, Basel, Switzerland). The obtained data were analyzed using the delta-Ct method.

### Antibodies

Anti-CD4 antibody (Clone: GK1.5 (FITC), SONY, Tokyo, Japan), anti-CD8a antibody (Clone: 53-6.7 (APC), SONY), anti-CD8b antibody (eBioH35-17.2 (PE), Invitrogen, CA, USA), anti-TCRβ antibody (Clone: H57-597 (FITC), SONY), anti-TCRβ antibody (Clone: H57-597 (APC/Cy7), BioLegend, CA, USA), anti-CD69 antibody (Clone: H1.2F3 (APC), BioLegend), anti-CD5 antibody (Clone: 53-7.3 (APC), BioLegend), anti-CD44 antibody (Clone: IM7 (Alexa Fluor 700), BioLegend), anti-CD62L antibody (Clone: MEL-14 (PE), BioLegend), anti-TCR Vβ5.1, 5.2 antibody (Clone: MR9-4 (PE), BioLegend), anti-TCR HY antibody (Clone: T3.70 (PE), eBioscience, CA, USA), anti-B220 antibody (Clone: RA3-6B2 (FITC), SONY), LEAF-purified anti-CD3ε antibody (Clone: 145-2C11, BioLegend), LEAF-purified anti-CD28 antibody (Clone: 37.51, BioLegend), Biotin anti-TCRβ antibody (Clone: H57-597, BioLegend) and Biotin anti-CD69 antibody (Clone: H1.2F3, BioLegend) were used in this study.

### Flow cytometry

The thymus and spleen were harvested to obtain single lymphocytes suspension. For splenocytes, red blood cells (RBCs) were lysed with RBC lysis buffer (0.015 M NH_4_Cl, 0.1 mM KHCO_3_, 0.01 mM Na_2_EDTA). Live cell numbers were counted by using Countess system (Thermo Fisher Scientific). The obtained samples (1 × 10^6^ cells) were then stained with anti-CD4 antibody (1:200), anti-CD8a antibody (1:100), anti-CD8b antibody (1:200), anti-TCRβ antibody (1:200 (FITC), 1:100 (APC/Cy7)), anti-CD69 antibody (1:100), anti-CD5 antibody (1:100), anti-CD44 antibody (1:200), anti-CD62L antibody (1:100), anti-TCR Vβ5.1, 5.2 antibody (1:100), anti-TCR HY antibody (1:200) or anti-B220 antibody (1:100) in 100 μl of FCM buffer (10% fetal bovine serum (FBS) in phosphate-buffered saline (PBS)) for >30 min at 4 °C in the dark. For IEL precursors and iNKT measurement, 1 × 10^6^ thymocytes were stained with α-GalCer-loaded CD1d tetramer (TS-MCD-1 (PE), MBL, Aichi, Japan) in 50 μl of FCM buffer for >30 min at 4 °C in the dark, and then stained with additional antibodies (see Supplementary Fig. 4 for the details). To analyze T_reg_ cells, the obtained single cell suspension (3–5 × 10^6^ cells) was stained using Foxp3 staining kit (Clone: FJK-16s, eBioscience, CA, USA), essentially according to the manufacturer’s instruction. Cell fixation was performed for 10 min. Samples were analyzed on EC800 (SONY) and FACS CantoII (BD Bioscience, NJ, USA). Gating strategies are shown in Supplementary Fig. 14.

### RNA-seq and bioinformatic analysis

Total RNAs were extracted from the thymus and spleen as described in the RNA isolation section. RNA-seq libraries were generated using the SureSelect Strand-Specific RNA Library Prep for Illumina (Agilent) according to the manufacturer’s instructions. Sequencing experiments were performed with Hiseq2500 (Illumina; Single End 36 bp). The obtained reads were mapped to the mouse genome mm9 using Illumina Eland with the default parameter setting. Uniquely aligned reads were retrieved allowing up to 2 bp mismatches, and the number of exon-mapped overlapping reads were counted. The obtained gene list with reads per million per a kilobase (RPKM) scores were shown in Supplementary Datas 2 and 3. To identify DEGs, we first focused on the well-annotated protein-coding genes. RPKM scores from two replicates were averaged, and the ratio Δ*E*^*BAB*^/WT were calculated. In this calculation, 1 was added to all averaged RPKM scores to ignore scores below “1”, and to make analyses more stringent. The obtained ratios were used to sort genes to find candidate DEGs, followed by statistical analyses (unpaired two-tailed Student’s *t*-test) and qPCR validation.

### Cell sorting

To purify pre- and post-selection thymocytes, single cell suspension (1–2 × 10^7^ cells) was stained with anti-TCRβ antibody (1:100 or 1:200) and anti-CD69 antibody (1:100 or 1:200) in 100 μl of FCM buffer for >30 min at 4 °C in the dark. The stained cells were then tenfold diluted and sorted using SH800 cell sorter system (SONY).

### Ex vivo thymocytes cell death assay

Totally, 1 × 10^5^ total thymocytes were cultured with DEX (10 nM), PMA (2 ng/ml) or ionomycin (1 μg/ml or 0.1 μg/ml) in 200 μl of RPMI1640 media (10% FBS, 1% penicillin streptomycin, 50 μM 2-mercaptoethanol, 1 × nonessential amino acids (nacalai tesque, Kyoto, Japan) and 1 × sodium pyruvate (nacalai tesque)) in a 96-well flat bottom plate. DMSO- or EtOH-treated cells served as controls. Viability was measured by Annexin V FLUOS staining kit (Roche) at days 0–2.

### Ex vivo TCR stimulation

Ex vivo TCR stimulation experiments were performed essentially as described previously^7,25^. Briefly, 12-well plates were coated with LEAF-purified anti-CD3ε and anti-CD28 antibodies (0 or 10 μg/ml in 500 μl of PBS, BioLegend) for 2 h at 37 °C. After 3 times-wash with 0.5 ml of PBS, 1 × 10^6^ cells of total thymocytes were cultured in 2 ml of DMEM media (10% FBS, 1% penicillin streptomycin, 2 mM l-glutamine, 50 μM 2-mercaptoethanol, 10 mM HEPES (pH 7.4), 1 × nonessential amino acids (nacalai tesque) and 1 × sodium pyruvate (nacalai tesque)) for 3 h (for qPCR experiments) or 9 h (for Annexin V and PI staining using Annexin V FLUOS staining kit (Roche)).

### Peptides

OVA_323–339_ peptide (ISQAVHAAHAEINEAGR, BEX, Tokyo, Japan), OVA_257–264_ peptide (SIINFEKL, BEX), gp33 (KAVYNFATC, BEX), and Q4R7 (SIIQFERL, BEX) were certified as >98% pure by HPLC. Peptides were dissolved in dimethyl sulfoxide (DMSO) at concentration of 100 mg/ml and stored at −80 °C. The peptide/DMSO solutions were diluted in PBS just before the experiment.

### OT-II negative selection assay

OT-II negative selection assay was performed as described previously^6^. Briefly, WT; OT-II^+^, *E*^*BAB*+/−^; OT-II^+^ and Δ*E*^*BAB*^; OT-II^+^ mice were injected intraperitoneally with 1 mg OVA_323–339_ peptide or OVA_257–264_ peptide as a control resuspended in 500 μl of PBS. Thymocytes were harvested after 72 h for analysis.

### OT-I tg fetal thymic organ culture

FTOC was performed as described previously^33–35^. Briefly, fetal thymic lobes were excised at embryonic day 15 (E15) and cultured on Whatman Nuclepore Track-Etched Membrane (WHA110409, GE Healthcare, Little Chalfont, England) floated on RPMI1640 media (10% FBS, 1% penicillin streptomycin, 50 μM 2-mercaptoethanol, 1 × nonessential amino acids (nacalai tesque, Kyoto, Japan) and 1 × sodium pyruvate (nacalai tesque)) in the presence of 2 μM OVA_257–264_, 2 μM Q4R7, or 20 μM gp33. On day 4, thymocytes were analyzed by flow cytometry.

### Immunoblotting assay

CD69^+^ and CD69^−^ T cells were enriched from thymocytes using biotinylated anti-CD69 antibody (BioLegend) and MojoSort Streptavidin Nanobeads (BioLegend). CD69^-^ T cells were further purified by depleting TCRβ^+^ cells using biotinylated anti-TCRβ antibody (BioLegend) and MojoSort Streptavidin Nanobeads (BioLegend). Expression of Bim and ACTB proteins in each subset was detected with anti-Bim antibody (1:1000, #2819, Cell Signaling, MA, USA) and anti-ACTB antibody (1:10,000, NB600-532, Novus Biologicals, CO, USA). As the secondary antibody, anti-rabbit IgG (1:15,000, 711-035-152, Jackson ImmumoResearch, PA, USA) was used. Signals were visualized with ECL plus Western Blotting Detection Reagents (GE Healthcare) and analyzed by the CCD digital imaging system LAS-4000 Luminescent Image Analyzer (GE Healthcare). Whole proteins were stained with SYPRO Ruby Protein Gel Stain (S12000, Thermo Fisher Scientific) and detected by the CCD digital imaging system LAS-4000 Luminescent Image Analyzer (GE Healthcare). Uncropped scans are available in the Source data file.

### Cytokine withdrawal assay

CD4^+^ or CD8^+^ T cells were isolated from splenocytes using MojoSort Mouse CD4 or CD8 T Cell Isolation Kit (BioLegend), respectively according to the manufacturer’s instruction. T cells were activated by culturing cells in RPMI1640 media (10% FBS, 1% penicillin streptomycin, 50 μM 2-mercaptoethanol, 1 × nonessential amino acids (nacalai tesque) and 1 × sodium pyruvate (nacalai tesque)) plus IL-2 (100 U/ml, Wako, Osaka, Japan) in 96 well flat bottom plate coated with LEAF-purified anti-CD3ε and anti-CD28 antibodies (5 μg/ml in 50 μl of PBS, BioLegend) for 48 hr. Activated T cells were then cultured for 1 day with RPMI1640 plus IL-2 (100 U/ml, Wako) in normal 96-well plate. After that, IL-2 was removed from the media and viability of CD4^+^ and CD8^+^ T cells was measured using Annexin V FLUOS staining kit (Roche) on days 1–3 after IL-2 withdrawal.

### Activation induced cell death assay

Isolation and activation of CD4^+^ or CD8^+^ T cells were performed as described in the “cytokine withdrawal assay” section. After activation and 1-day culture in the presence of IL-2, T cells were further incubated in RPMI1640 media (10% FBS, 1% penicillin streptomycin, 50 μM 2-mercaptoethanol, 1 × nonessential amino acids (nacalai tesque) and 1 × sodium pyruvate (nacalai tesque)) without IL-2 in a 96-well flat bottom plate coated with LEAF-purified anti-CD3ε (5 μg/ml in 50 μl of PBS, BioLegend) for 6 h. Cell viability was determined using Annexin V FLUOS staining kit (Roche).

### Histochemistry

Mouse tissues were fixed in 10% neutral buffered formalin. Samples were then processed into 6 micron sections, mounted, and Hematoxylin and eosin (H&E)-stained (Genostaff, Tokyo, Japan). The prepared samples were observed with Nikon Ni-E microscope (Nikon, Tokyo, Japan).

### Experimental autoimmune encephalomyelitis

Experimental autoimmune encephalomyelitis (EAE) experiments were performed essentially as described previously^47^. Briefly, mice (WT, Δ*E*^*BAB*^, and *Bim*^*−/*^^−^) were subjected to subcutaneous injection at two different sites with an emulsion of complete Freund’s adjuvant (CFA; 250 μg heat-killed *Mycobacterium tuberculosis* H37Ra (Difco) dissolved in 50 μl incomplete Freund’s adjuvant (Sigma)) and 250 μg myelin oligodendrocyte glycoprotein (MOG) peptides (amino acids 35–55) in 50 μl PBS. Mice were then injected with 200 ng of *Pertussis Toxin* (Calbiochem) intraperitoneally at day 0 and day 2 after EAE induction. The clinical scores were assessed as follows: (1) flaccid tail, (2) impaired righting reflex and/or gait, (3) partial hind limb paralysis, (4) total hind limb paralysis, and (5) total hind limb paralysis with partial forelimb paralysis.

### Statistics

The sample size was chosen as follows. First, the number of animals was minimized as much as possible in light of animal ethics. Second, against effect size estimated in each experiment, ≥80–90% power was favored. Third, in most cases, *n* = 5 was set as a threshold according to the previous report^55^. These three criterion functioned to determine the sample size as *n* = 5–8 in most experiments. Moreover, dot-plot/box plot representation of data provided insights into how the samples were distributed, and thus into the extent of difference of two groups. This, in the specific cases, led us to conclude that relatively small number of animals (i.e., less than five animals) was enough to support our conclusion.

According to the observed differences, our sample size appeared to be appropriate to meet≥80–90% power. In addition, dot-plot/box plot representation of data was useful to estimate sample distribution. Clearly abnormal distribution was not observed in our experiments, further supporting that our statistical tests were appropriate.

Significant differences between two groups were examined using one or two-tailed, unpaired *t* test, or Mann–Whitney *U* test. One-tailed test was chosen when we had hypothesis and/or previous knowledge regarding direction of changes (e.g., increased or decreased) in experiments.

### Reporting summary

Further information on research design is available in the Nature Research Reporting Summary linked to this article.

## Supplementary information

Supplementary Information

Peer Review File

Reporting Summary

Description of Additional Supplementary Files

Supplementary Data 1

Supplementary Data 2

Supplementary Data 3

Source Data

## Data Availability

The RNA-seq data have been deposited in DNA Data Bank of Japan (DDBJ) under the accession code DRA004726 and DRA008123. Source data underlying graphs and blots are supplied as a Source data file. All other data are included in the supplemental information or available from the authors upon reasonable requests.
