# Supplementary material for: Identification of a genomic enhancer that enforces proper apoptosis induction in thymic negative selection
Source: Nat Commun. 2019 Jun 13;10:2603. doi: 10.1038/s41467-019-10525-1 (PMC6565714; doi:10.1038/s41467-019-10525-1)
Supplement: Supplementary file 1 — Supplementary Information [file 41467_2019_10525_MOESM1_ESM.pdf]

Supplementary information

Identification of a genomic enhancer that enforces proper apoptosis induction in thymic negative selection

Miki Arai Hojo[1,2], Kyoko Masuda[3], Hiroaki Hojo[2,3,4], Yosuke Nagahata[3], Keiko Yasuda[3], Daiya Ohara[3], Yusuke Takeuchi[3], Keiji Hirota[3], Yutaka Suzuki[1], Hiroshi Kawamoto[3], Shinpei Kawaoka[2,3,4,*]

[1]Graduate School of Frontier Science, The University of Tokyo, Kashiwa-shi, Chiba, Japan

[2]The Thomas N. Sato BioMEC-X Laboratories, Advanced Telecommunications Research Institute International (ATR), Soraku-gun, Kyoto, Japan

[3]Institute for Frontier Life and Medical Sciences, Kyoto University, Kyoto-shi, Kyoto, Japan

[4]ERATO Sato Live Bio-forecasting Project, Japan Science and Technology Agency (JST), Soraku-gun, Kyoto, Japan

[*]Corresponding Author:
Shinpei Kawaoka, Ph.D.
Institute for Frontier Life and Medical Sciences
Kyoto University
53 Shogoin-kawahara-cho, Sakyo-ku, Kyoto 606-8507, Japan
TEL: +81-75-751-4804
FAX: +81-75-751-3839
Email: kawaokashinpei@gmail.com

Supplementary Figure 1

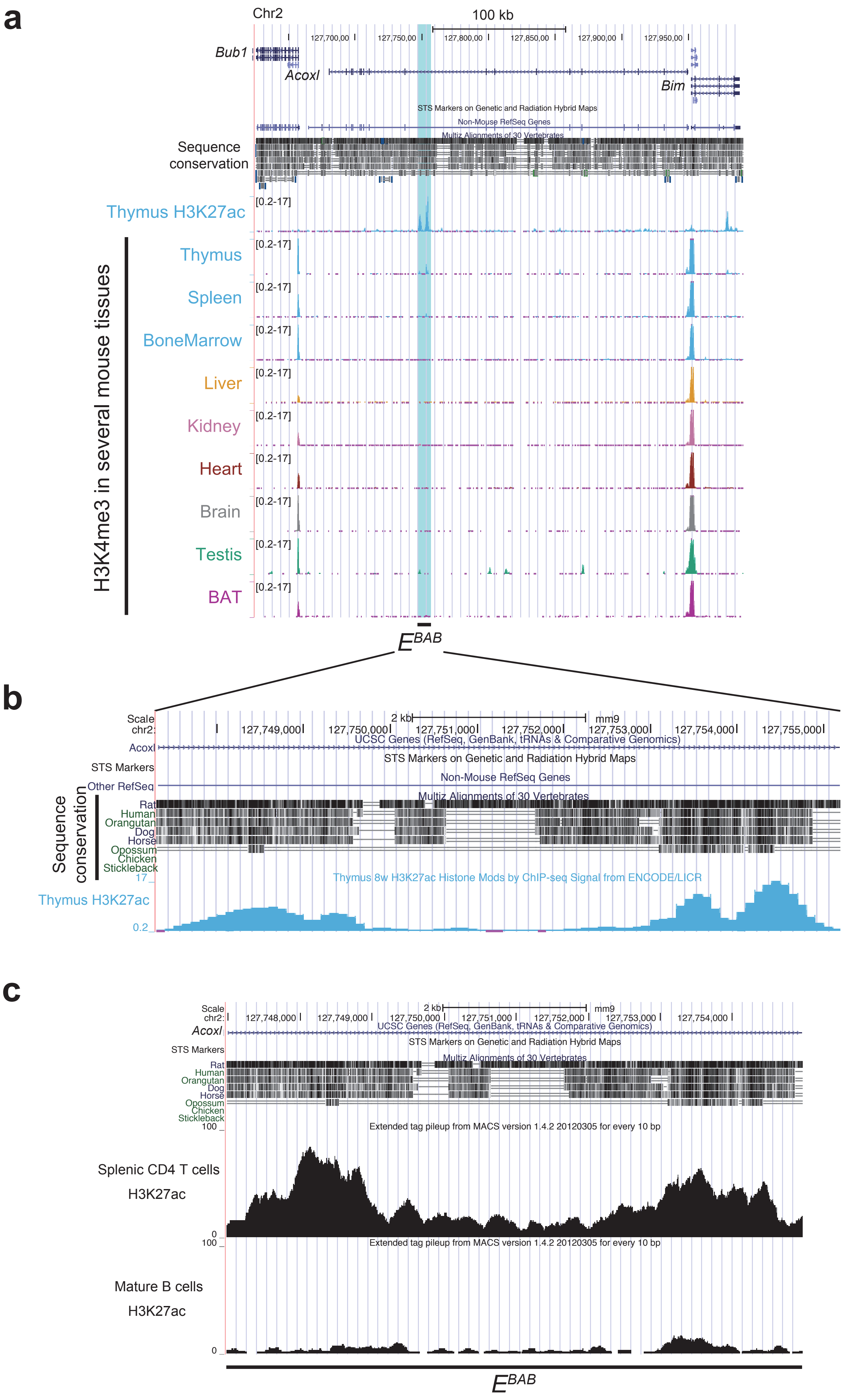

Supplementary Figure 1: H3K4me3 profiles, sequence conservation, and H3K27ac profiles of E^{BAB} in splenic CD4 T cells and mature B cells.
(a) H3K4me3 ChIP-seq profiles obtained from the UCSC genome browser (mm9). H3K27ac ChIP-seq profile from the thymus is also shown. The indicated tissues are thymus, spleen, bone marrow, liver, kidney, heart, brain, testis, and brown adipose tissues (BAT).
(b) The enlarged image of sequence conservation between mouse and others (including human) in E^{BAB}. H3K27ac ChIP-seq profile from the thymus is also shown.
(c) H3K27ac ChIP-seq data from GSE67443 (splenic CD4 T cells) and GSE60005 (mature B cells) were visualized. The raw sequence data were retrieved from Gene Expression Omnibus (GEO) database to generate wig format files using MACS peak calling, and then wig files were uploaded to the browser.

Supplementary Figure 2

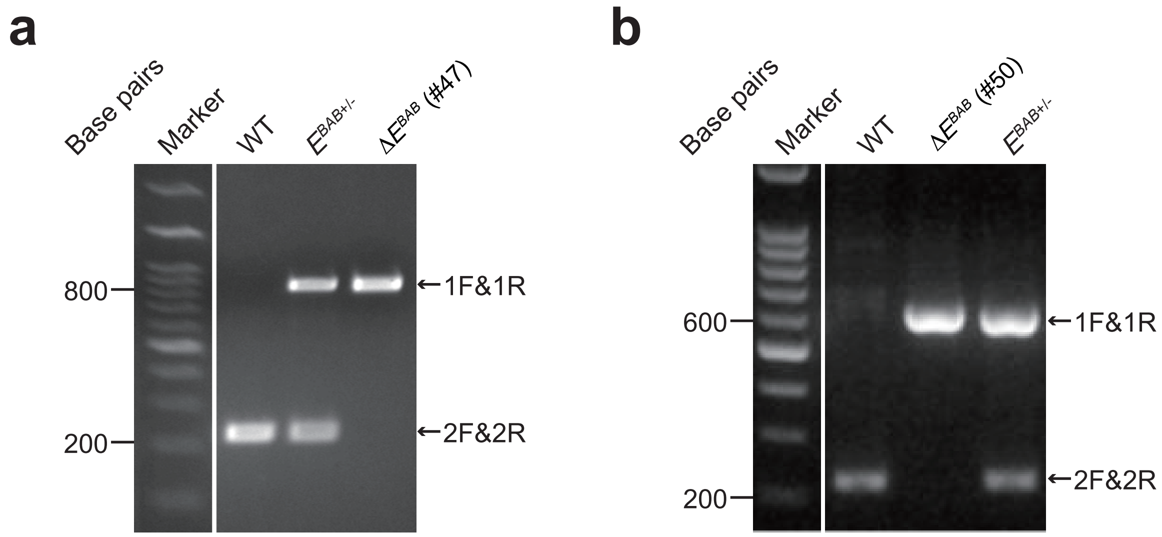

Supplementary Figure 2: Genomic PCR to characterize the alleles from founder #47 and #50. **(a-b)** Genomic PCR amplifying WT and E^{BAB} knockout allele are shown for #47 **(a)** and #50 **(b)**. For primer infomartion see also Fig. 1c and Supplementary Data 1.

Supplementary Figure 3

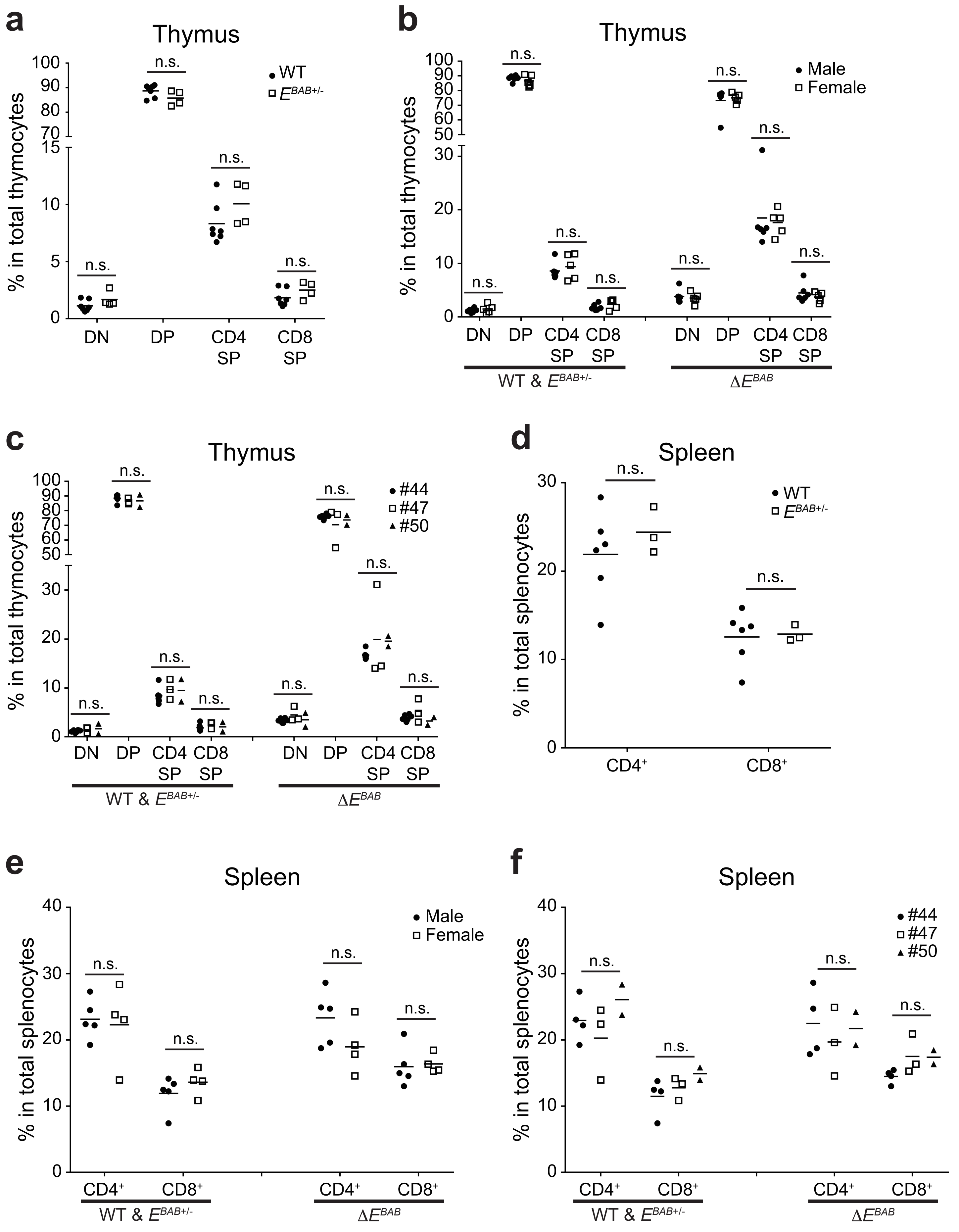

Supplementary Figure 3: Comparison on T cell phenotypes between WT and $E^{BAB+/-}$, males and females, and the three founders.

DN, DP, CD4 SP and CD8 SP proportions in the thymus were quantified. $CD4^{+}$ and $CD8^{+}$ proportions in the spleen were also analyzed.

(a-c) Comparison on WT and $E^{BAB+/-}$ **(a)**, males and females **(b)**, and the three founders **(c)** in the thymus.

(d-f) Comparison on WT and $E^{BAB+/-}$ **(d)**, males and females **(e)**, and the three founders **(f)** in the spleen.

Data are pooled from 11 independent experiments (**a**; n = 7 WT mice, n = 4 $E^{BAB+/-}$ mice, **b**; n = 6 male mice, n = 5 female mice, **c**; n = 6 #44 mice, n = 3 #47 mice, n = 2 #50 mice) or 9 independent experiments (**d**; n = 6 WT mice, n = 3 $E^{BAB+/-}$ mice, **e**; n = 5 male mice, n = 4 female mice, **f**; n = 4 #44 mice, n = 3 #47 mice, n = 2 #50 mice). Each symbol represents an individual mouse; small horizontal lines indicate the mean. No statistically significant differences were detected ($P \geq 0.05$; unpaired two-tailed Student's *t*-test **(a, b, d, e)**, one-way ANOVA **(c, f)**).

Supplementary Figure 4

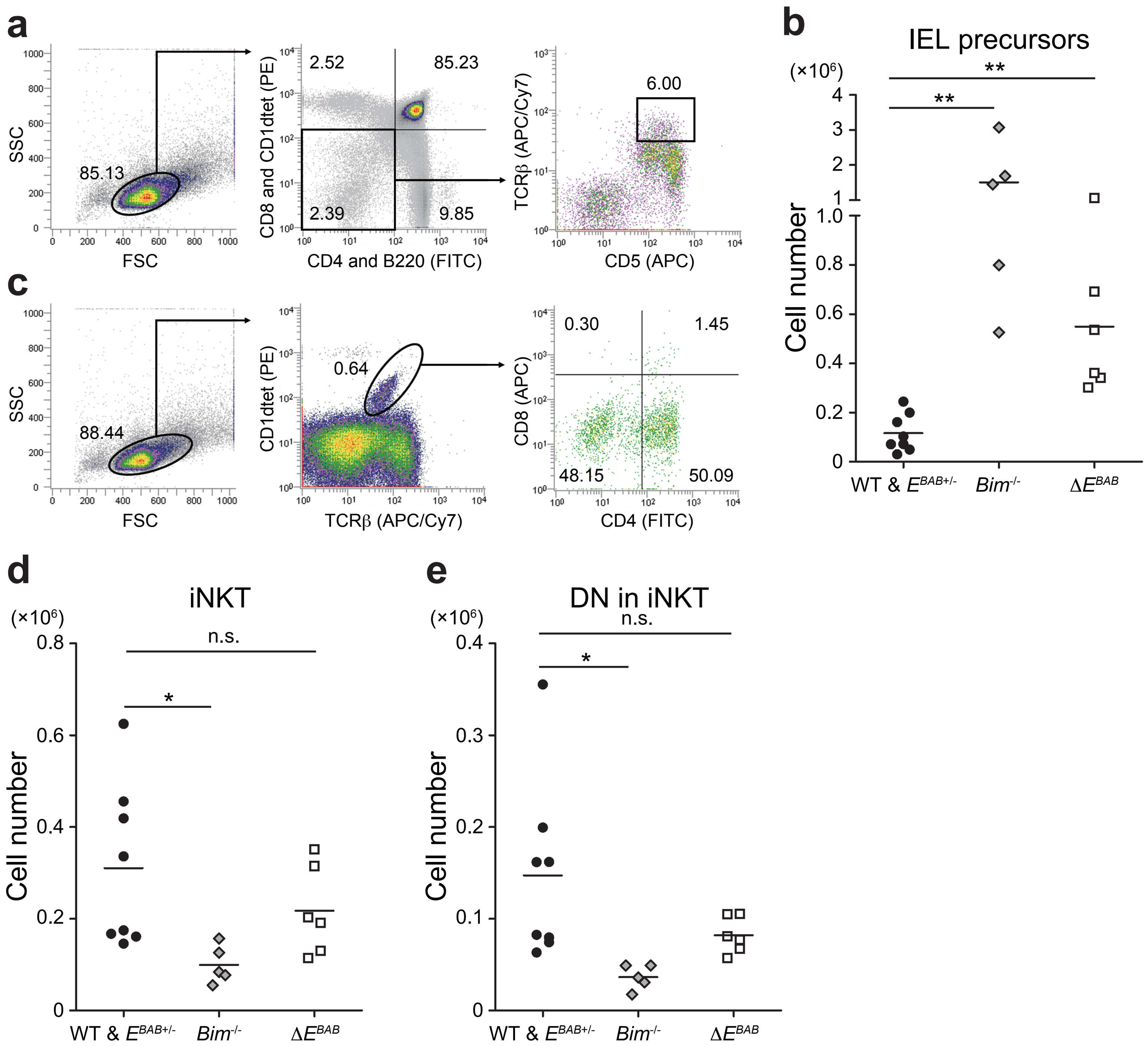

Supplementary Figure 4: Effects of *E*^{BAB} knockouts on IEL precursors and iNKT cells in the thymus.

(a) Gating strategy for IEL precursors (DN TCRαβ^{+}CD5+ thymocytes). The number in the plot is representative percentage of each gate. SSC side scatter, FSC forward scatter.

(b) The number of IEL precursors (DN TCRαβ^{+}CD5+ thymocytes).

(c) Gating strategy for iNKT (CD1d tetramer^{+}TCRαβ^{+} thymocytes). The number in the plot is representative percentage of each gate. SSC side scatter, FSC forward scatter.

(d-e) The number of total **(d)** and DN **(e)** iNKT (CD1d tetramer^{+}TCRαβ^{+} thymocytes).

Data are representative of **(a, c)** or pooled from **(b, d, e)** 7 independent experiments (n = 3 WT & *E*$^{BAB+/-}$–Δ*E*^{BAB} littermate pairs, n = 1 WT–*Bim*$^{-/-}$ littermate pair, n = 3 sex-matched WT–*Bim*$^{-/-}$–Δ*E*^{BAB} trios, n = 1 sex-matched WT–*Bim*$^{-/-}$ pair, 9-17-weeks-old). Each symbol **(b, d, e)** represents an individual mouse; small horizontal lines indicate the mean. n.s. not significant ($P \geq 0.05$); *$P < 0.05$, **$P < 0.01$, ***$P < 0.001$ (unpaired two-tailed Student's *t*-test).

Supplementary Figure 5

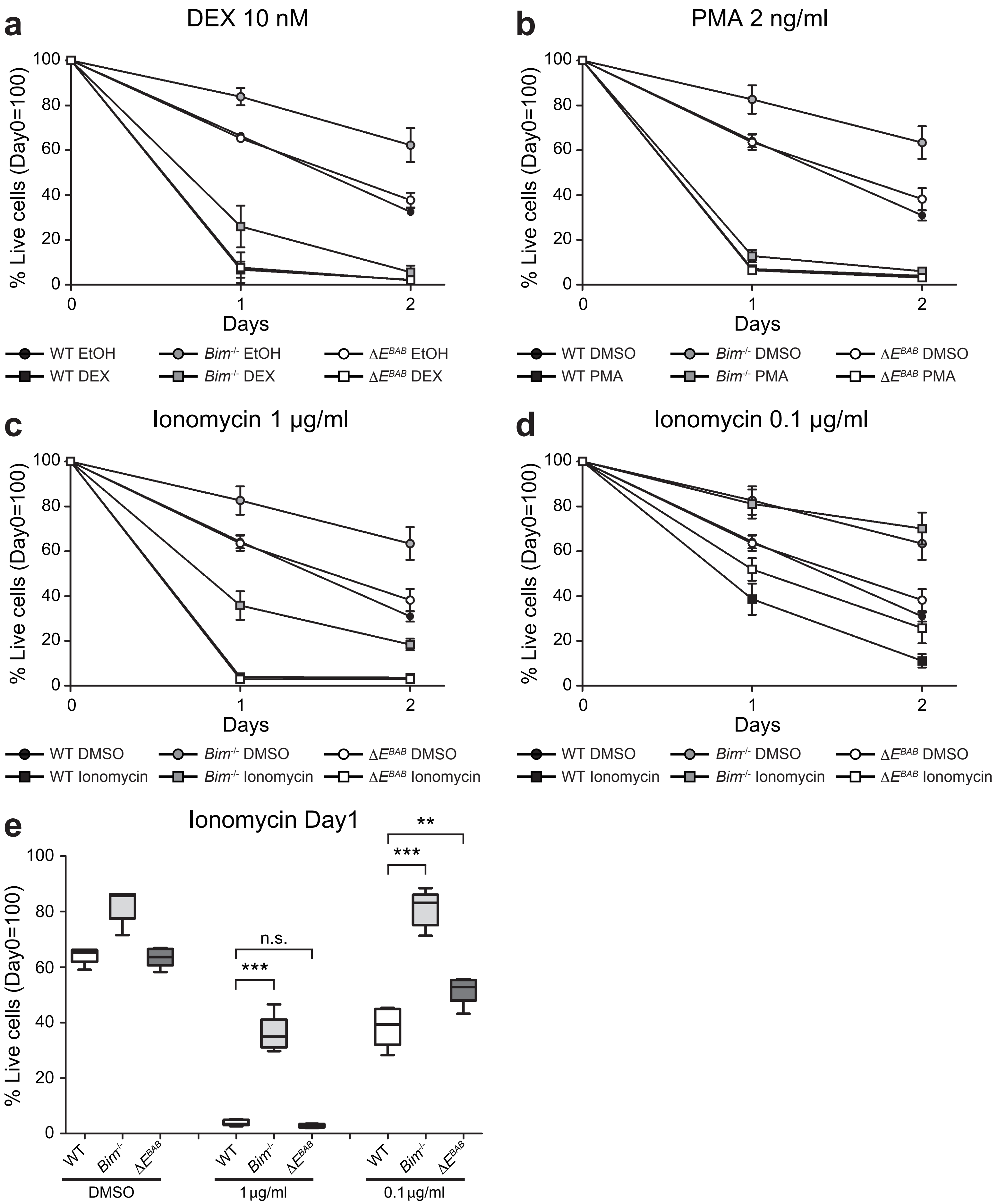

Supplementary Figure 5: Effects of E^{BAB} deletion on thymocytes cell death induced by a series of apoptotic stimuli *ex vivo*.

(a-d) Viability (% Annexin $V^{-}PI^{-}$) of thymocytes treated with 10 nM DEX **(a)**, 2 ng/ml PMA **(b)**, 1 μg/ml ionomycin **(c)**, 0.1 μg/ml ionomycin **(d)**. Data are pooled from 6 independent experiments (n = 5 sex-matched WT–$Bim^{-/-}$–ΔE^{BAB} trios, 5-26-weeks-old, mean ± s.d.).

(e) Viability (% Annexin $V^{-}PI^{-}$) of thymocytes treated with ionomycin on day 1.

Data are pooled from 6 independent experiments (n = 5 sex-matched WT–$Bim^{-/-}$–ΔE^{BAB} trios, 5-26-weeks-old). Edges of the box are the 25th and 75th percentiles, and error bars extend to the maximum and minimum. Outliers are defined as the data point that is located outside of q3 + 1.5(q3 − q1) and q1 – 1.5(q3 − q1), in which q1 and q3 are the 25th and 75th percentiles. n.s. not significant ($P \geq 0.05$); $^{**}P < 0.01$, $^{***}P < 0.001$ (unpaired two-tailed Student's *t*-test).

Supplementary Figure 6

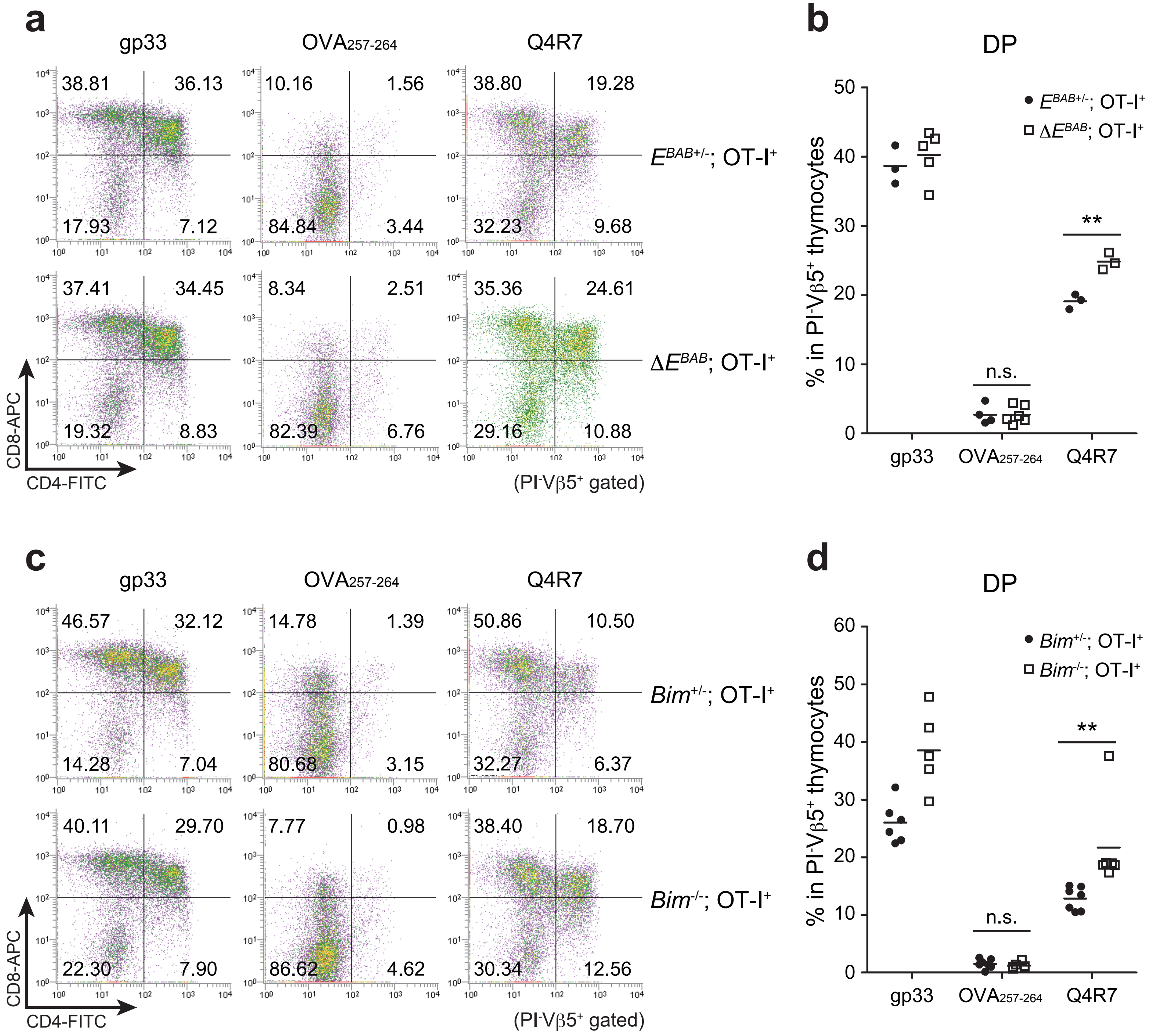

Supplementary Figure 6: Deletion of E^{BAB} rescues thymocytes from negative selection in a TCR affinity-dependent manner in the OT-I FTOC model.

(a) CD4 versus CD8 flow cytometric analysis of PI^-TCR $V\beta5^+$ thymocytes from *$E^{BAB+/-}$*; OT-I^+ or Δ*E^{BAB}*; OT-I^+ FT treated with 20 μM gp33, 2 μM $OVA_{257\text{-}264}$, or 2 μM Q4R7 peptides. The number in the plot is representative percentage of each gate.

(b) DP thymocyte proportion of PI^-TCR $V\beta5^+$ thymocytes from *$E^{BAB+/-}$*; OT-I^+ or Δ*E^{BAB}*; OT-I^+ FT treated as in Supplementary Fig. 6a.

(c) CD4 versus CD8 flow cytometric analysis of PI^-TCR $V\beta5^+$ thymocytes from *$Bim^{+/-}$*; OT-I^+ or *$Bim^{-/-}$*; OT-I^+ FT treated as in Supplementary Fig. 6a.

(d) DP thymocyte proportion of PI^-TCR $V\beta5^+$ thymocytes from *$Bim^{+/-}$*; OT-I^+ or *$Bim^{-/-}$*; OT-I^+ FT treated as in Supplementary Fig. 6a.

Data are representative of **(a, c)** or pooled from **(b, d)** 4 independent experiments (n = 3 *$E^{BAB+/-}$*; OT-I^+, n = 5 Δ*E^{BAB}*; OT-I^+, n = 6 *$Bim^{+/-}$*; OT-I^+ and n = 5 *$Bim^{-/-}$*; OT-I^+ for gp33 treatment, n = 4 *$E^{BAB+/-}$*; OT-I^+, n = 6 Δ*E^{BAB}*; OT-I^+, n = 7 *$Bim^{+/-}$*; OT-I^+ and n = 6 *$Bim^{-/-}$*; OT-I^+ for $OVA_{257\text{-}264}$ treatment, and n = 3 *$E^{BAB+/-}$*; OT-I^+, n = 3 Δ*E^{BAB}*; OT-I^+, n = 7 *$Bim^{+/-}$*; OT-I^+ and n = 6 *$Bim^{-/-}$*; OT-I^+ for Q4R7 treatment, embryonic day 15). Each symbol **(b, d)** represents an individual lobe; small horizontal lines indicate the mean. n.s. not significant ($P \geq 0.05$); $^*P < 0.05$, $^{**}P < 0.01$ (unpaired two-tailed Student's *t*-test for Δ*E^{BAB}* and unpaired two-tailed Student's *t*-test or Mann-Whitney U test for *$Bim^{-/-}$*).

Supplementary Figure 7

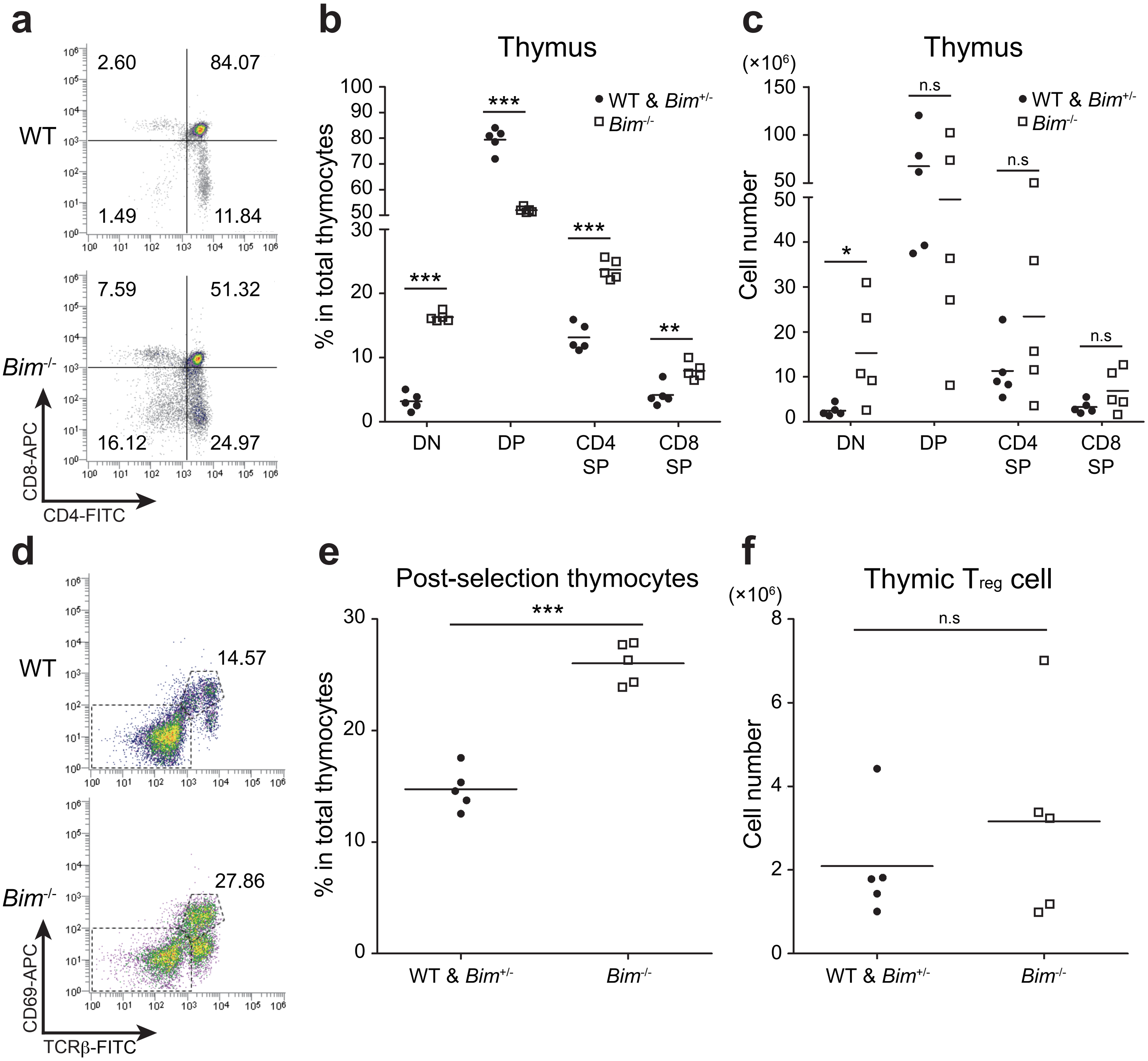

Supplementary Figure 7: Thymic T cell phenotypes in *Bim* KO mice.

(a) Flow cytometric analysis of CD4 versus CD8 thymosyte populations. The number in the plot is representative percentage of each gate.

(b) DN, DP, CD4 SP and CD8 SP thymocyte proportions.

(c) DN, DP, CD4 SP and CD8 SP thymocyte cell numbers.

(d) Flow cytometric analysis of TCRβ versus CD69 thymocyte populations. The number in the plot is representative percentage of the gate.

(e) Post-selection ($TCR\beta^{high}CD69^{high}$) thymocyte proportion.

(f) $CD4^{+}Foxp3^{+}$ thymic T_{reg} cell numbers.

Data are representative of **(a, d)** or pooled from **(b, c, e, f)** 5 independent experiments (n = 5 WT & *Bim*$^{+/-}$–*Bim*$^{-/-}$ littermate pairs, 7-17-weeks-old). Each symbol represents an individual mouse; small horizontal lines indicate the mean. *$P < 0.05$, **$P < 0.01$, ***$P < 0.001$ (unpaired one-tailed Student's *t*-test).

Supplementary Figure 8

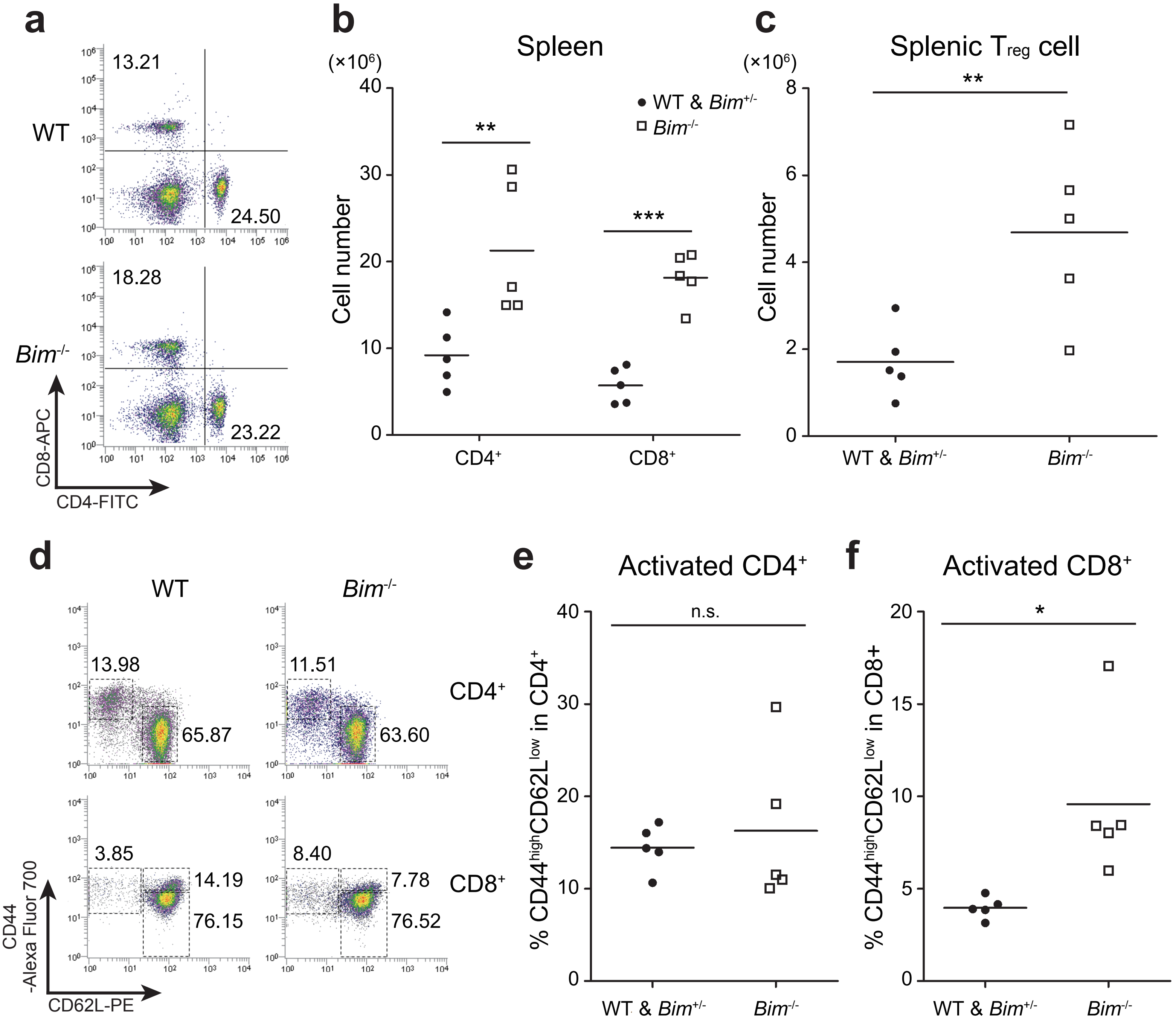

Supplementary Figure 8: Splenic T cell phenotypes in *Bim* KO mice.

(a) Flow cytometric analysis of CD4 versus CD8 T cell populations in the spleen. The number in the plot is representative percentage of each gate.

(b) $CD4^+$ and $CD8^+$ splenic T cell numbers.

(c) $CD4^+Foxp3^+$ splenic Treg cell numbers.

(d) Flow cytometric analysis of CD62L versus CD44 populations in $CD4^+$ or $CD8^+$ splenic T cell. The number in the dot plot is representative percentage of each gate.

(e-f) Activated $CD4^+$ **(e)** and $CD8^+$ **(f)** proportions.

Data are representative of **(a, d)** or pooled from **(b, c, e, f)** 5 independent experiments (n = 5 WT & $Bim^{+/-}$–$Bim^{-/-}$ littermate pairs, 7-17-weeks-old). Each symbol represents an individual mouse; small horizontal lines indicate the mean. *$P < 0.05$, **$P < 0.01$, ***$P < 0.001$ (unpaired one-tailed Student's *t*-test).

Supplementary Figure 9

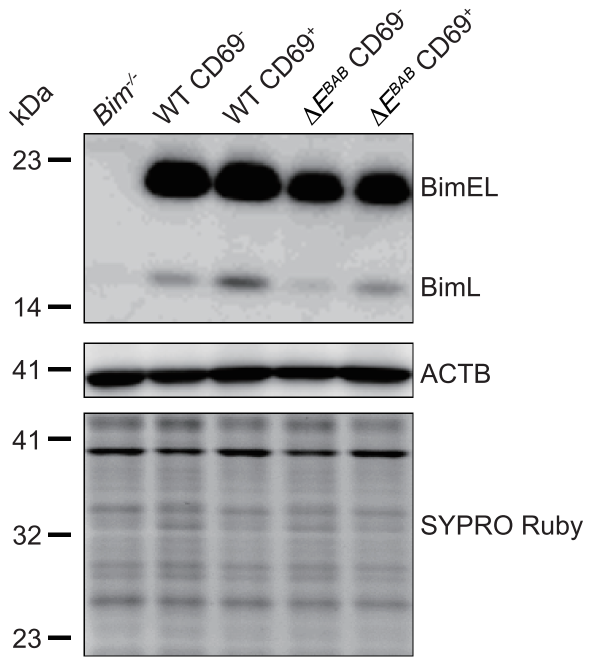

Supplementary Figure 9: Western blot analysis for Bim proteins. Western blotting for Bim and ACTB proteins in CD69$^-$ and CD69$^+$ thymocytes of WT and Δ*E*^{BAB} mice. SYPRO Ruby staining is also shown as a loading control. Total thymocytes from *Bim* KO mice were used as a control.

Supplementary Figure 10

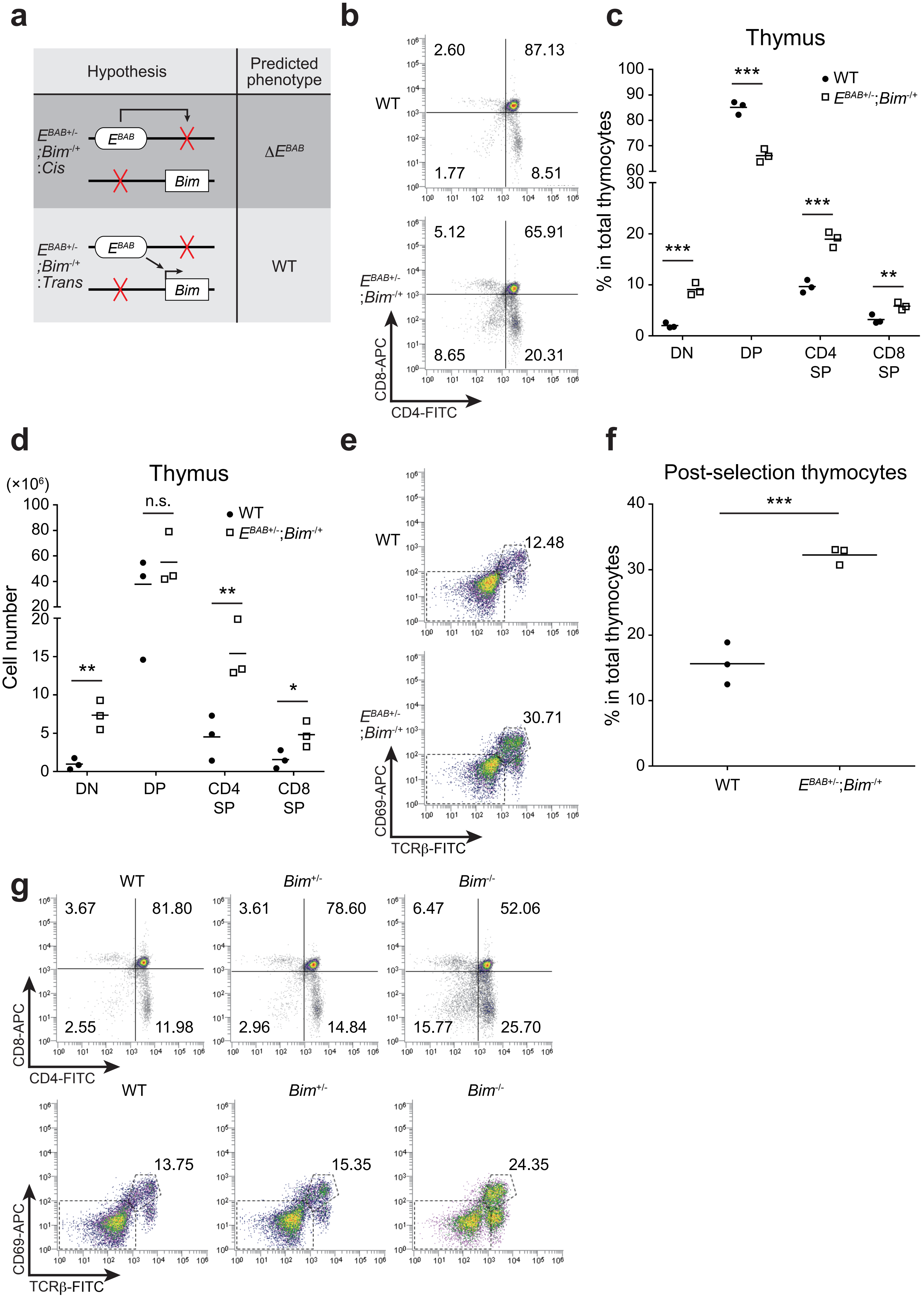

Supplementary Figure 10: E^{BAB} regulates *Bim* in *cis*.

(a) Schematic representation of hypothesis and predicted phenotype.

(b) Flow cytometric analysis of CD4 versus CD8 thymosyte populations. The number in the plot is representative percentage of each gate.

(c) DN, DP, CD4 SP and CD8 SP thymocyte proportions.

(d) DN, DP, CD4 SP and CD8 SP thymocyte cell numbers.

(e) Flow cytometric analysis of TCRβ versus CD69 thymocyte populations. The number in the plot is representative percentage of the gate.

(f) Post-selection (TCRβ^{high}CD69^{high}) thymocyte proportion.

(g) Flow cytometric analysis of CD4 versus CD8 and TCRβ versus CD69 thymocyte population of WT, *Bim*$^{+/-}$ and *Bim*$^{-/-}$ mice. The number in the plot is percentage of each gate.

Data are representative of **(b, e)** or pooled from **(c, d, f)** 3 independent experiments (n =3 sex-matched WT–ΔE^{BAB} pairs, 8-10-weeks-old). Data in **(g)** are representative of 3 independent experiments (n = 2 WT mice, n = 1 *Bim*$^{+/-}$ mice, 7-8-weeks-old). Each symbol represents an individual mouse; small horizontal lines indicate the mean. unpaired two-tailed Student's *t*-test, *$P < 0.05$, **$P < 0.01$, ***$P < 0.001$.

Supplementary Figure 11

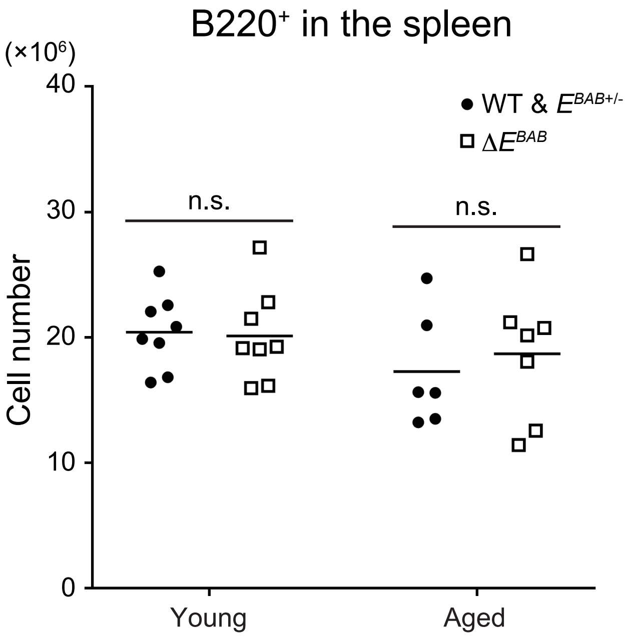

Supplementary Figure 11: ΔE^{BAB} does not affect B cells in the spleen.

B220^{+} splenocyte cell numbers in young and aged mice.

Data are pooled from 8 independent experiments (Young; n = 8 WT & $E^{BAB+/-}$–ΔE^{BAB} littermate pairs, 7-17-weeks-old) or 5 independent experiments (Aged; n = 6 WT & $E^{BAB+/-}$ mice, n = 7 ΔE^{BAB} mice, WT & $E^{BAB+/-}$–ΔE^{BAB} littermate pair or trio, 30-36-weeks-old). Each symbol represents an individual mouse; small horizontal lines indicate the mean. No statistically significant differences were detected ($P \geq 0.05$; unpaired two-tailed Student's *t*-test).

Supplementary Figure 12

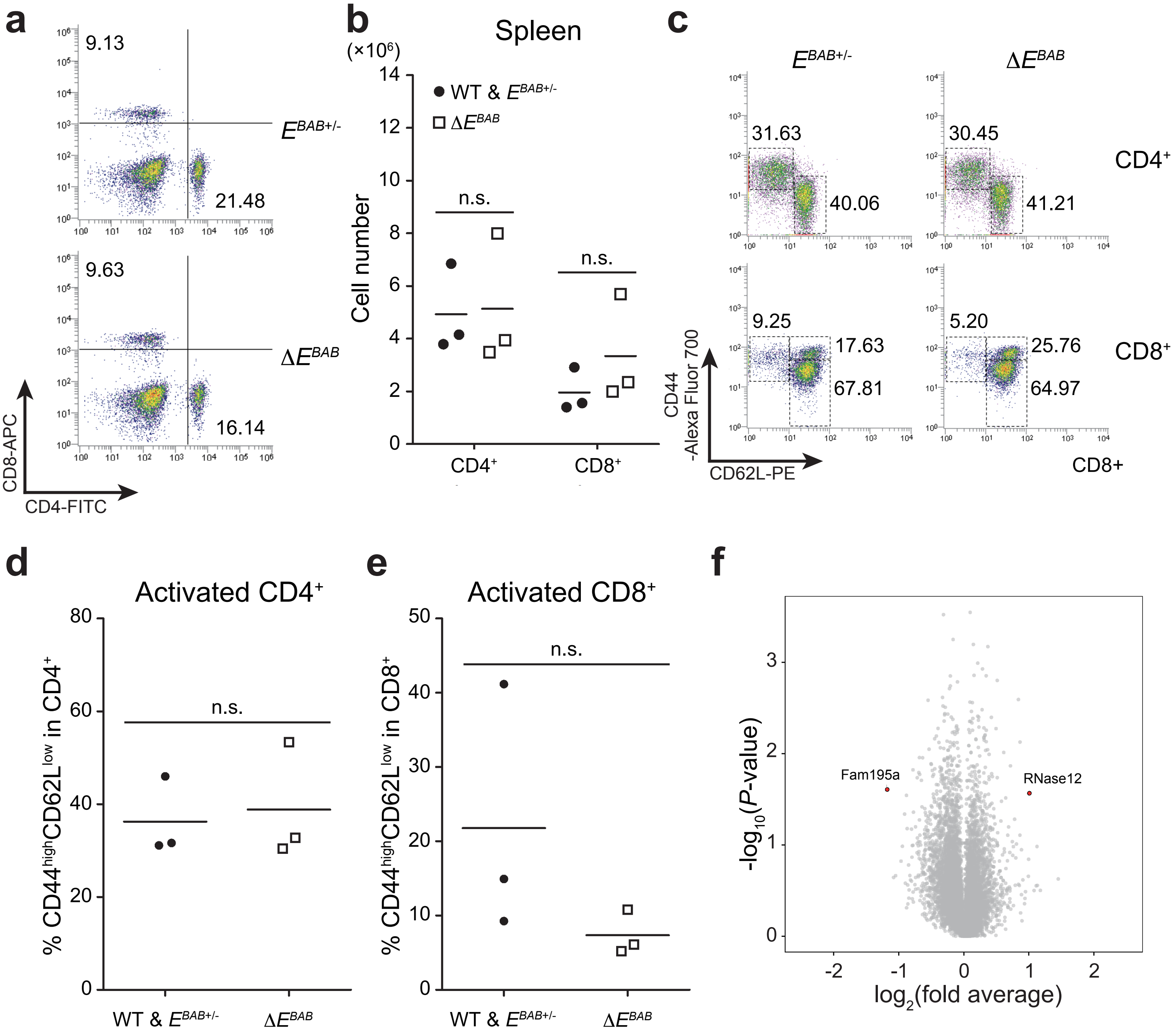

Supplementary Figure 12: ΔE^{BAB} does not affect peripheral T cells in the spleen.

(a) Flow cytometric analysis of CD4 vs CD8 T cell populations in the spleen. The number in the plot is representative percentage of each gate.

(b) $CD4^+$ and $CD8^+$ splenic T cell numbers.

(c) Flow cytometric analysis of CD62L versus CD44 populations in $CD4^+$ or $CD8^+$ splenic T cell. The number in the plot is representative percentage of each gate.

(d-e) Activated $CD4^+$ **(d)** and $CD8^+$ **(e)** proportions of aged mice.

Data are representative of **(a, c)** or pooled from **(b, d, e)** 3 independent experiments (n = 3 WT & $E^{BAB+/-}$–ΔE^{BAB} littermate pairs, 50-51-weeks-old)

Each symbol **(b, d, e)** represents an individual mouse; small horizontal lines indicate the mean. n.s. not significant ($P \geq 0.05$) (unpaired two-tailed Student's *t*-test).

(f) Scatter plot of genes showing $\log_2$ fold change versus $-\log_{10}$ *P*-value between WT and ΔE^{BAB} analyzed by RNA-seq (n = 2 WT–ΔE^{BAB} littermate pairs, 10-11-weeks-old). Two candidate DEGs showing more than 2-fold change with $P < 0.05$ are highlighted (unpaired two-tailed Student's *t*-test).

Supplementary Figure 13

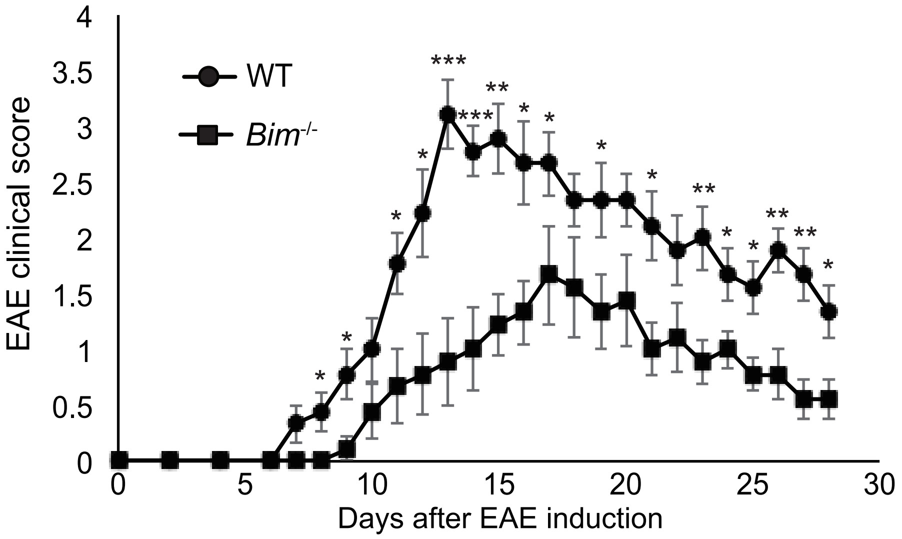

Supplementary Figure 13: Effects of *Bim* on EAE.

The mean (±s.e.m.) clinical scores at the days after EAE was induced in WT (control) (n = 9) and *Bim*$^{-/-}$ mice (n = 9). The incidence of EAE: control 9/9, *Bim*$^{-/-}$ 9/9. unpaired two-tailed Student's *t*-test, $^{*}P < 0.05$, $^{**}P < 0.01$, $^{***}P < 0.001$.

Supplementary Figure 14

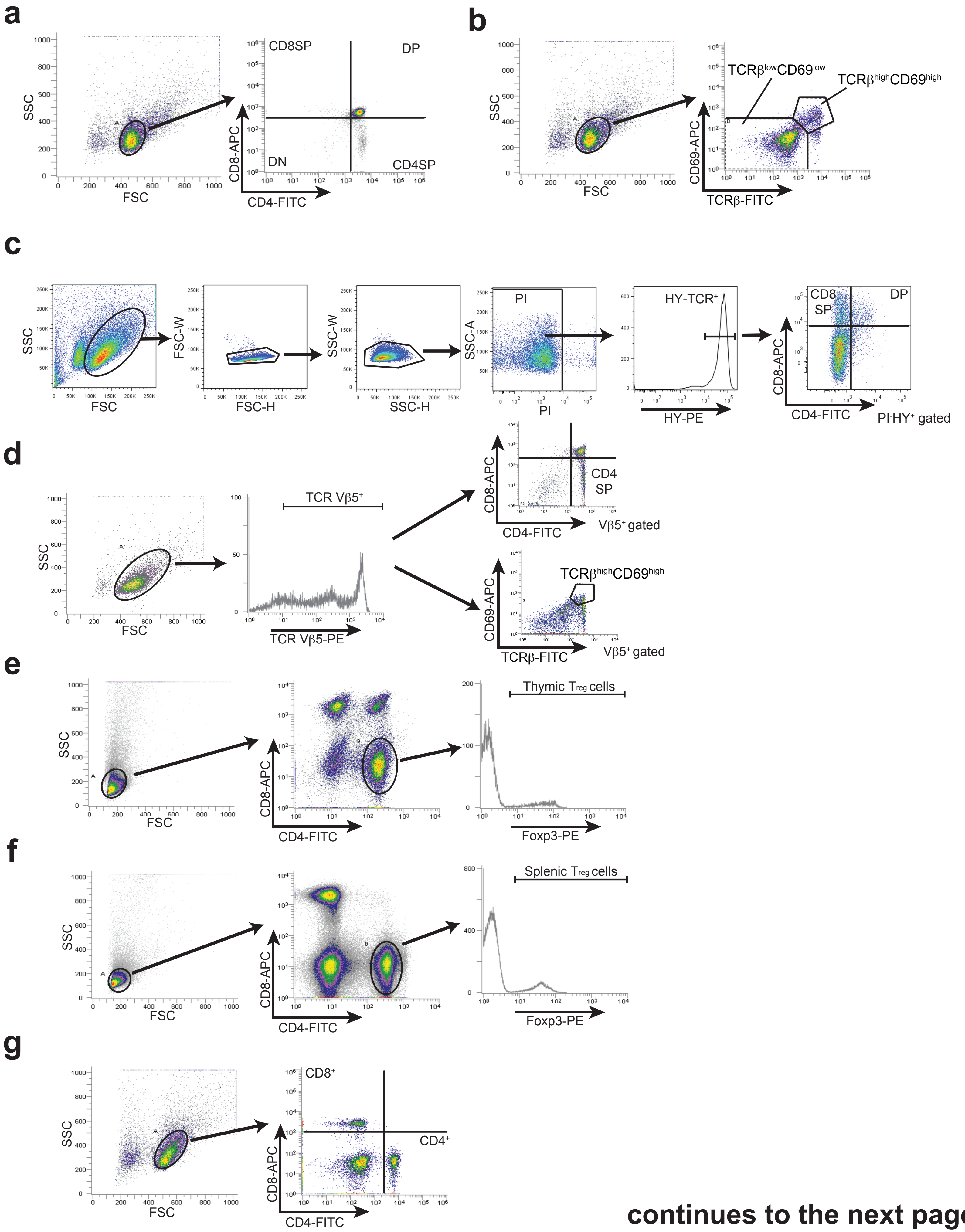

continues to the next page

Supplementary Figure 14, continued

h

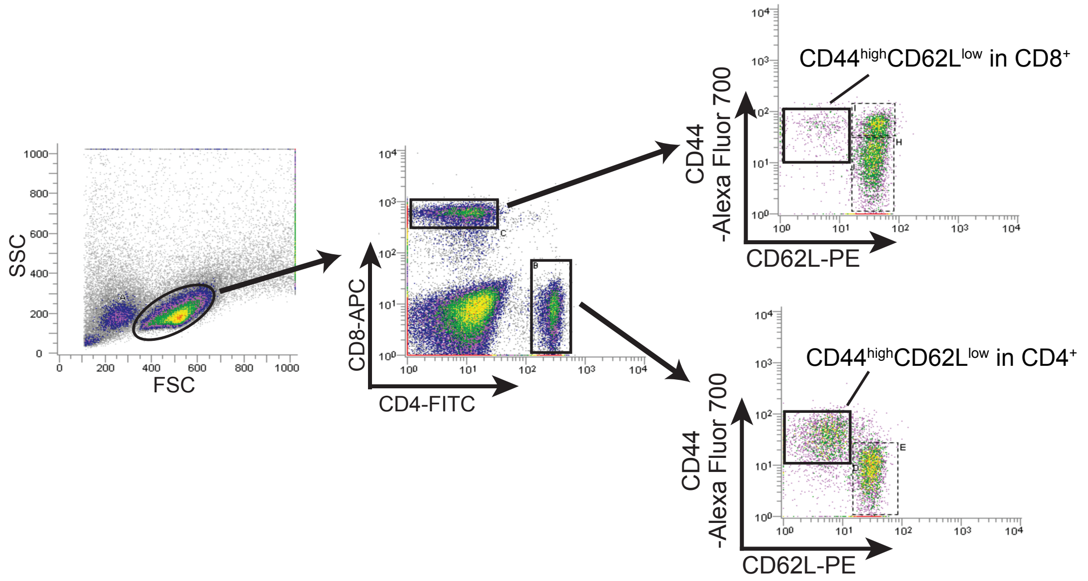

i

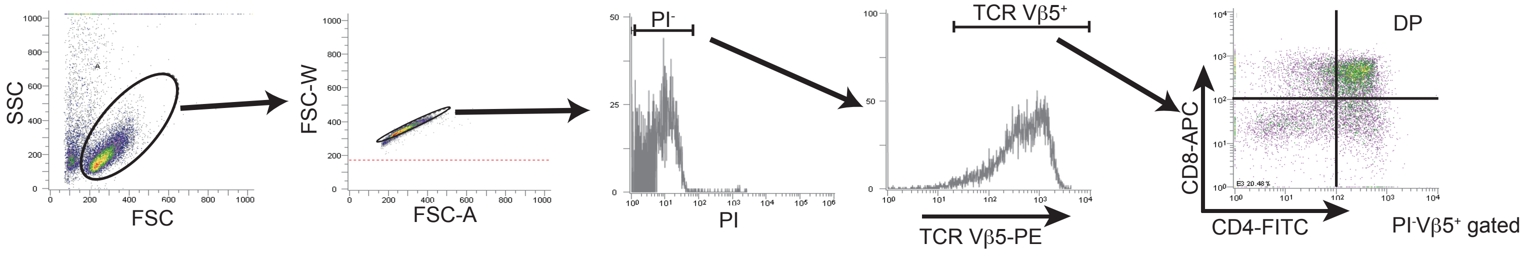

j

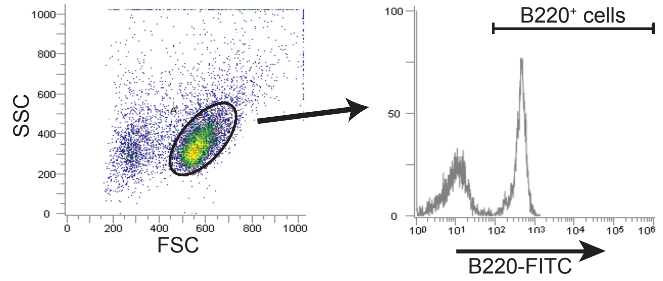

Supplementary Figure 14: Gating strategies used in this study.

Gating strategies to sort **(a)** DN, DP, CD4SP, and CD8SP thymocytes (Fig. 2a-d, Supplementary Fig.3a-c, Supplementary Fig. 7a-c, Supplementary Fig. 10b-d,10g), **(b)** pre- and post-selection thymocytes (Fig. 2f-h, Supplementary Fig. 7d-e, Supplementary Fig. 10e-f), **(c)** HY-TCR$^+$ thymocytes (Fig. 3b-f), **(d)** OT-II TCR$^+$ thymocytes (Fig. 4), **(e)** thymic T_{reg} cells (Fig. 6a, Supplementary Fig. 7f), **(f)** splenic T_{reg} cells (Fig. 6b, Supplementary Fig. 8c), **(g)** splenic CD4$^+$ and CD8$^+$ T cells (Fig. 6c-d, Supplementary Fig. 8a-b, Supplementary Fig. 12a-b), **(h)** activated T cells in the spleen (Fig. 8a-c, Supplementary Fig. 8d-f, Supplementary Fig. 12c-e), **(i)** OT-I TCR$^+$ thymocytes (Supplementary Fig. 6), and **(j)** B220$^+$ T cells in the spleen (Supplementary Fig. 11).
