# Supplementary material for: Identification of a genomic enhancer that enforces proper apoptosis induction in thymic negative selection
Source: Nat Commun. 2019 Jun 13;10:2603. doi: 10.1038/s41467-019-10525-1 (PMC6565714; doi:10.1038/s41467-019-10525-1)
Supplement: Supplementary file 3 — Reporting Summary [file 41467_2019_10525_MOESM3_ESM.pdf]

nature research

Corresponding author(s): Shinpei Kawaoka
Last updated by author(s): Apr 2, 2019

Reporting Summary

Nature Research wishes to improve the reproducibility of the work that we publish. This form provides structure for consistency and transparency in reporting. For further information on Nature Research policies, see Authors & Referees and the Editorial Policy Checklist.

Statistics

For all statistical analyses, confirm that the following items are present in the figure legend, table legend, main text, or Methods section.

n/a	Confirmed	
☐	☒	The exact sample size (n) for each experimental group/condition, given as a discrete number and unit of measurement
☐	☒	A statement on whether measurements were taken from distinct samples or whether the same sample was measured repeatedly
☐	☒	The statistical test(s) used AND whether they are one- or two-sided<br>*Only common tests should be described solely by name; describe more complex techniques in the Methods section.*
☒	☐	A description of all covariates tested
☐	☒	A description of any assumptions or corrections, such as tests of normality and adjustment for multiple comparisons
☐	☒	A full description of the statistical parameters including central tendency (e.g. means) or other basic estimates (e.g. regression coefficient) AND variation (e.g. standard deviation) or associated estimates of uncertainty (e.g. confidence intervals)
☐	☒	For null hypothesis testing, the test statistic (e.g. F, t, r) with confidence intervals, effect sizes, degrees of freedom and P value noted<br>*Give P values as exact values whenever suitable.*
☒	☐	For Bayesian analysis, information on the choice of priors and Markov chain Monte Carlo settings
☒	☐	For hierarchical and complex designs, identification of the appropriate level for tests and full reporting of outcomes
☒	☐	Estimates of effect sizes (e.g. Cohen's d, Pearson's r), indicating how they were calculated

Our web collection on statistics for biologists contains articles on many of the points above.

Software and code

Policy information about availability of computer code

Data collection	Flow cytometry data were collected with EC800 analyzer analysis software and BD FACS Diva. RNA-seq data were obtained by Illumina Hiseq platform.
Data analysis	For flow cytometry analysis, EC800 analyzer analysis software, BD FACS Diva and FlowJo (V. 10) were used. For other data analyses, microsoft excel and graphpad PRISM were used.

For manuscripts utilizing custom algorithms or software that are central to the research but not yet described in published literature, software must be made available to editors/reviewers. We strongly encourage code deposition in a community repository (e.g. GitHub). See the Nature Research guidelines for submitting code & software for further information.

Data

Policy information about availability of data

All manuscripts must include a data availability statement. This statement should provide the following information, where applicable:

- Accession codes, unique identifiers, or web links for publicly available datasets
- A list of figures that have associated raw data
- A description of any restrictions on data availability

RNA-seq data that support the findings of this study have been deposited in DNA Data Bank of Japan (DDBJ) with the accession code DRA004726 and DRA008123.

Field-specific reporting

Please select the one below that is the best fit for your research. If you are not sure, read the appropriate sections before making your selection.

☒ Life sciences ☐ Behavioural & social sciences ☐ Ecological, evolutionary & environmental sciences

For a reference copy of the document with all sections, see nature.com/documents/nr-reporting-summary-flat.pdf

Life sciences study design

All studies must disclose on these points even when the disclosure is negative.

Sample size	The sample size was chosen as follows. First, the number of animals was minimized as much as possible in light of animal ethics. Second, against effect size estimated in each experiment, ≥ 80%-90% power was favored. Third, in most cases, n = 5 was set as a threshold according to the previous report (Krzywinski, M. & Altman, N. Points of significance: Comparing samples-part I. Nat Methods 11, 215-216 (2014)). These three criterion functioned to determine the sample size as n = 5-8 in most experiments. Moreover, dot-plot representation of data provided insights into how the samples were distributed, and thus into the extent of difference of two groups. This, in the specific cases, led us to conclude that relatively small number of animals (i.e. less than 5 animals) was enough to support our conclusion.
Data exclusions	No data exclusions were performed.
Replication	All experiments have been performed several times using multiple mice. Different methods, transgenic mice models, and ex vivo systems, were used to support our findings together.
Randomization	All experiments were performed without randomization. In most cases, e.g. a KO mouse was analyzed with its littermate, and/or sex-matched control, side-by-side on the same day.
Blinding	Blinding was performed in the injection process of OT-II negative selection assay. In other experiments, blinding was not done.

Reporting for specific materials, systems and methods

We require information from authors about some types of materials, experimental systems and methods used in many studies. Here, indicate whether each material, system or method listed is relevant to your study. If you are not sure if a list item applies to your research, read the appropriate section before selecting a response.

Materials & experimental systems

n/a	Involved in the study	
☐	☒	Antibodies
☒	☐	Eukaryotic cell lines
☒	☐	Palaeontology
☐	☒	Animals and other organisms
☒	☐	Human research participants
☒	☐	Clinical data

Methods

n/a	Involved in the study	
☒	☐	ChIP-seq
☐	☒	Flow cytometry
☒	☐	MRI-based neuroimaging

Antibodies

Antibodies used	Anti-Bim antibody (#2819S, Cell Signaling) and anti-ACTB antibody (NB600-532, Novus Biologicals) were used for immunoblotting assay. Anti-CD4 antibody (Clone: GK1.5 (FITC), SONY, Tokyo, Japan), anti-CD8a antibody (Clone: 53-6.7 (APC), SONY), anti-CD8b antibody (eBioH35-17.2 (PE), Invitrogen, CA, USA), anti-TCRβ antibody (Clone: H57-597 (FITC), SONY), anti-TCRβ antibody (Clone: H57-597 (APC/Cy7), BioLegend, CA, USA), anti-CD69 antibody (Clone: H1.2F3 (APC), BioLegend), anti-CD5 antibody (Clone: 53-7.3 (APC), BioLegend), anti-CD44 antibody (Clone: IM7 (Alexa Fluor 700), BioLegend), anti-CD62L antibody (Clone: MEL-14 (PE), BioLegend), anti-TCR Vβ5.1, 5.2 antibody (Clone: MR9-4 (PE), BioLegend), anti-TCR HY antibody (Clone: T3.70 (PE), eBioscience, CA, USA), anti-B220 antibody (Clone: RA3-6B2 (FITC), SONY), LEAF-purified anti-CD3ε antibody (Clone: 145-2C11, BioLegend), LEAF-purified anti-CD28 antibody (Clone: 37.51, BioLegend), Biotin anti-TCRβ antibody (Clone: H57-597, BioLegend) and Biotin anti-CD69 antibody (Clone: H1.2F3, BioLegend) were used in this study.
Validation	These antibodies have been validated e.g. in mouse cell lines by manufactures. We also validated anti-Bim antibody using Bim KO sample as a control.

Animals and other organisms

Policy information about studies involving animals; ARRIVE guidelines recommended for reporting animal research

Laboratory animals	Mouse

Wild animals	N.A.
Field-collected samples	N.A.
Ethics oversight	All animal protocols were approved by the Animal Care and Use committee of Advanced Telecommunications Research Institute International (permission numbers: AN20140002, AN20150002, AN20160002, AN20170002, and AN20180002) and by the committee of Kyoto university (permission numbers: L18-1, L18-1-2, and L19-2).

Note that full information on the approval of the study protocol must also be provided in the manuscript.

Flow Cytometry

Plots

Confirm that:

☒ The axis labels state the marker and fluorochrome used (e.g. CD4-FITC).

☒ The axis scales are clearly visible. Include numbers along axes only for bottom left plot of group (a 'group' is an analysis of identical markers).

☒ All plots are contour plots with outliers or pseudocolor plots.

☒ A numerical value for number of cells or percentage (with statistics) is provided.

Methodology

Sample preparation	The thymus and spleen harvested from mice were cut into several smaller pieces in a petri dish containing RPMI1640 medium. The obtained pieces were homogenized using a slide glass, or 100um cell strainer and plunger of 1mL syringe, and single cell suspension was obtained. Single cell suspension was collected into a tube and washed several times with fresh medium. For the spleen, erythrocytes were hemolyzed. After washing, large debris and cell clumps were removed from the sample using 70um cell strainer.
Instrument	Sony EC 800 was used for analysis described in Fig. 2a-d, f-g, Fig. 3a, Fig. 4, Fig. 6, Fig. 7a-b, g-h, Fig. 8a-c, Supplementary Fig. 3-8, 10-12. BD FACS CantoII was used for analysis described in Fig. 3b-f. Sony SH800 was used for sorting experiments in Fig. 2h, Fig. 5e-h.
Software	Data were collected with EC800 analyzer analysis software and BD FACS Diva.<br>Obtained data were analyzed using EC800 analyzer analysis software and FlowJo (V.10).
Cell population abundance	Abundance of the relevant cell populations was ascertained by a post-sort purity check or qPCR experiments for surface marker genes such as CD69 against at least one representative sample. Post-sort fractions were 70-95 % depending on experiments as determined by a post-sort purity check. Regarding qPCR-based validations, for example, expression of CD69 in the sorted fraction was > 6 times higher than that in the negative fraction.
Gating strategy	The cells were gated on FSC/SSC basis on the location known to contain lymphoid cells. Unstained and control sample were used to determine the gates.

☒ Tick this box to confirm that a figure exemplifying the gating strategy is provided in the Supplementary Information.
