# Supplementary material for: Identification of a genomic enhancer that enforces proper apoptosis induction in thymic negative selection
Source: Nat Commun. 2019 Jun 13;10:2603. doi: 10.1038/s41467-019-10525-1 (PMC6565714; doi:10.1038/s41467-019-10525-1)
Supplement: Supplementary file 4 — Description of Additional Supplementary Files [file 41467_2019_10525_MOESM4_ESM.pdf]

Description of Additional Supplementary Files

File Name: Supplementary Data 1
Description: Primer and sgRNA oligo sequences used in this study.

File Name: Supplementary Data 2
Description: RPKM scores from the WT and KO thymus.

File Name: Supplementary Data 3
Description: RPKM scores from the WT and KO spleen.
