# Supplementary material for: Identification of a genomic enhancer that enforces proper apoptosis induction in thymic negative selection
Source: Nat Commun. 2019 Jun 13;10:2603. doi: 10.1038/s41467-019-10525-1 (PMC6565714; doi:10.1038/s41467-019-10525-1)
Supplement: Supplementary file 2 — Peer Review File [file 41467_2019_10525_MOESM2_ESM.pdf]

Editorial Note: This manuscript has been previously reviewed at another journal that is not operating a transparent peer review scheme. This document only contains reviewer comments and rebuttal letters for versions considered at Nature Communications . Mentions of prior referee reports have been redacted.

REVIEWERS' COMMENTS:

Reviewer #1 (Remarks to the Author):

The authors have inserted all the new data that I have requested and they have made all the text changes that I have recommended. I do not have any further requests.

Reviewer #2 (Remarks to the Author):

The authors' revisions have helped clarify their findings and solidified the conclusion that the Ebab enhancer is important for expression of Bim and clonal deletion during thymic negative selection, but appears to be dispensable for peripheral tolerance. In these ways the report is valuable for showing that Bim expression is controlled through distinct mechanisms at successive stages of T cell maturation and tolerance induction.
